# Insulin-like growth factor-1 stimulates regulatory T cells and suppresses autoimmune disease

**DOI:** 10.15252/emmm.201303376

**Published:** 2014-11-03

**Authors:** Daniel Bilbao, Luisa Luciani, Bjarki Johannesson, Agnieszka Piszczek, Nadia Rosenthal

**Affiliations:** 1 Mouse Biology Unit, European Molecular Biology Laboratory (EMBL)Monterotondo, Italy; 2 National Heart and Lung Institute, Imperial CollegeLondon, UK; 3 Australian Regenerative Medicine Institute/EMBL Australia, Monash UniversityClayton, Vic., Australia

**Keywords:** autoimmunity, diabetes, IGF-1, multiple sclerosis, T regulatory cells

## Abstract

The recent precipitous rise in autoimmune diseases is placing an increasing clinical and economic burden on health systems worldwide. Current therapies are only moderately efficacious, often coupled with adverse side effects. Here, we show that recombinant human insulin-like growth factor-1 (rhIGF-1) stimulates proliferation of both human and mouse regulatory T (Treg) cells *in vitro* and when delivered systemically via continuous minipump, it halts autoimmune disease progression in mouse models of type 1 diabetes (STZ and NOD) and multiple sclerosis (EAE) *in vivo*. rhIGF-1 administration increased Treg cells in affected tissues, maintaining their suppressive properties. Genetically, ablation of the IGF-1 receptor specifically on Treg cell populations abrogated the beneficial effects of rhIGF-1 administration on the progression of multiple sclerotic symptoms in the EAE model, establishing a direct effect of IGF-1 on Treg cell proliferation. These results establish systemically delivered rhIGF-1 as a specific, effective stimulator of Treg cell action, underscoring the clinical feasibility of manipulating natural tolerance mechanisms to suppress autoimmune disease.

## Introduction

The immune system protects adult mammals against pathogens while restricting those responses to avoid harm to the host. Active suppression of inflammation and immune responses by regulatory T (Treg) cells is essential in maintaining this equilibrium and immunological self-tolerance (O'Garra & Vieira, 2004; Littman & Rudensky, 2010). Treg cell-based therapies have held great promise for restoring tolerance in autoimmune diseases (Maloy & Powrie, 2001; Wing & Sakaguchi, 2010). Yet efforts to obtain sufficient cell numbers for transplant, or to develop effective strategies for boosting endogenous Treg cell function, have met with limited success.

We have previously implicated IGF-1 as a powerful enhancer of regenerative responses in multiple tissue types (Musaro *et al*, 2004; Santini *et al* 2007, Semenova *et al* 2010). However, cumulative evidence points to an additional role of the IGF-1/IGF-1R signaling pathway in regulating the immune response (van Buul-Offers & Kooijman, 1998; Smith, 2010). In previous work using autoimmune models, IGF-1 was primarily considered as a pleiotropic factor with mitogenic properties that acted mainly on the affected tissues, enabling their repair while protecting them from the stress of an immune attack (Smith *et al*, 1991; Yao *et al*, 1995; Liu *et al*, 1997; Agudo *et al*, 2008). However, to date, IGF-1-based therapies for selected autoimmune diseases have not been effective (Lovett-Racke *et al*, 1998; Cannella *et al*, 2000; Genoud *et al*, 2005).

Here, we define the Treg cell as a direct target of IGF-1 action in both mouse and human and demonstrate the more general ability of IGF-1 to control pathological responses in mouse models of autoimmune disease, using a clinically relevant continuous delivery protocol based on intensive systemic recombinant human IGF-1 release. These findings establish the efficacy of systemically delivered rhIGF-1, an FDA-approved therapeutic, in neutralizing autoimmune attack through local Treg cell recruitment, underscoring the broad applicability of intensive rhIGF-1 therapy in the treatment of autoimmune disorders.

## Results

### rhIGF-1 stimulates proliferation of human and mouse Treg cells *ex vivo*

**Figure 1:**
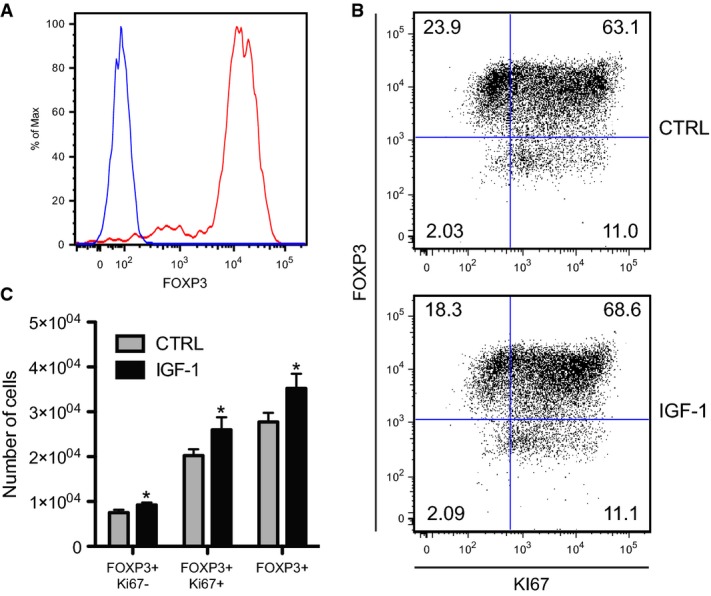
rhIGF stimulates the proliferation of human Treg cells *in vitro* FOXP3 expression levels of the sorted human Treg population used in the *in vitro* assay (red) compared to isotype-labeled cells (blue) showing the purity (> 98%) of the Treg cells used in the *in vitro* proliferation assay.Representative flow cytometric analysis of treated (rhIGF-1) and control (untreated) human Treg cells stained with Foxp3 and Ki67 antibodies after 6 days of *in vitro* culture. Control and treated cells were stimulated with anti-CD3 and CD28 antibodies.Flow cytometric analysis of the stimulated and control human Treg cells showing increased Treg cell number after 6 days of culture with rhIGF-1. [*P(FOXP3^+^Ki67^−^) = 0.0273; *P*(FOXP3^+^Ki67^+^) = 0.0492; *P*(FOXP3^+^) = 0.0393; *n* = 2].

To assess the potential effects of rhIGF-1 on Treg cell populations, Treg (CD4^+^ CD25^+^ CD127^low^) cells were FACS-sorted from human peripheral blood and analyzed for the transcription factor forkhead box P3 (Foxp3), a marker for Treg cells, which was expressed by > 98% of the sorted population (Fig 1A, Supplementary Fig S1A). When treated with commercially available rhIGF-1, human Treg cells showed increased proliferation and FOXP3 expression compared to untreated cells (Fig 1B and C), underscoring the therapeutic relevance of this simple protocol to promote Treg cell proliferation *ex vivo*.

**Figure 2:**
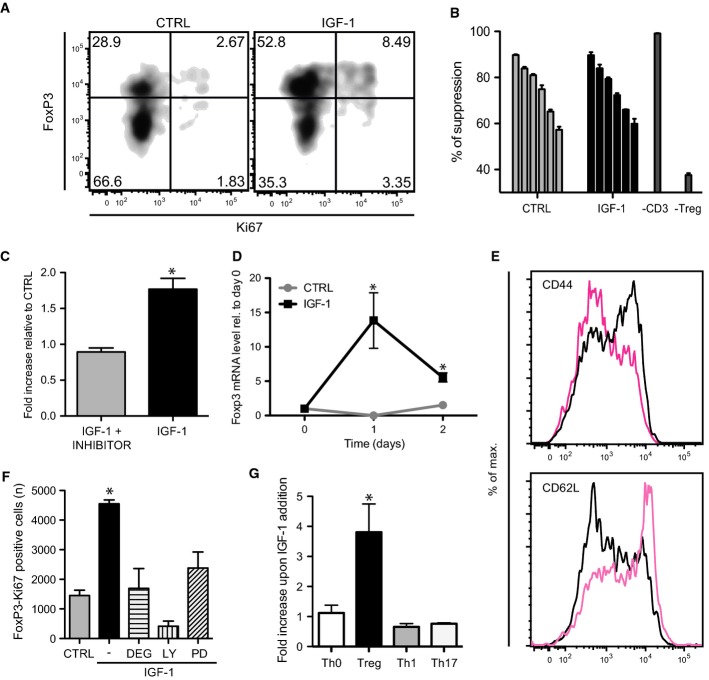
rhIGF-1 stimulates murine Treg cell proliferation *in vitro* FoxP3 and Ki67 flow cytometric analysis of purified mouse CD25^+^ splenic cells shows increased Treg cells after 2 days stimulation with rhIGF-1. In all *in vitro* experiments, cells were stimulated with anti-CD3 and CD28 antibodies.Naive murine Treg cells retain their ability to suppress T effector (Teff) cell proliferation *in vitro* after rhIGF-1 treatment (ratios Treg:Teff, from left to right 1:2, 1:4, 1:8, 1:16, 1:32, 1:64; *P* = 0.869; *n* = 3). (See also Supplementary Fig S1C).IGF receptor inhibitor (2 μM) blocks IGF-1-mediated expansion of murine Treg cells *in vitro* (*P = 0.0011; *n* = 3).Foxp3 qRT-PCR analysis of sorted murine CD4^+^CD25^+^ cells after the indicated times of rhIGF-1 stimulation shows increase in Foxp3 transcripts compared to untreated controls (*P_day1_ = 0.0379; *P_day2_ = 0.0132; *n* = 2).Representative flow cytometric analyses of surface expression markers (CD44 and CD62L) induced by rhIGF-1 on the Treg containing (CD4^+^CD25^+^) subset reflecting murine Treg cell activation.Flow cytometric analysis showing the effect of inhibitors of AKT (Deguelin, 1 μM; Deg), PI3-kinase (Ly-294,002, 10 μM; LY) and MAPK (PD.98,059, 10 μM; PD) on the proliferation of murine Treg cells after 2-day treatment with rhIGF-1 (*P = 0.0059; *n* = 2).rhIGF-1 expands the Treg cell subset (FoxP3^+^) but not CD4^+^CD25^−^ (Th0) or pro-inflammatory murine subsets Th1 (IFN-γ^+^) and Th17 (IL-17^+^), which remained unchanged after 2 days polarization and 3-day incubation with rhIGF-1 (*P = 0.0268; *n* = 3).

As in the human cell experiment, a stimulatory role for rhIGF-1 in mouse Treg cell function was documented *in vitro* by robust proliferation and FoxP3 activation in FACS-purified Treg cells treated with rhIGF-1 (Fig 2A and Supplementary Fig S1B). Whereas in humans, Foxp3 is not directly correlated with T-cell-mediated suppression (Wang *et al*, 2007), in the mouse, it is both necessary and sufficient for Treg development and function (Sakaguchi *et al*, 2007). Importantly, mouse Treg cells stimulated with rhIGF-1 retained their ability to suppress T effector cell proliferation (Fig 2B and Supplementary Fig S1C), while the anti-apoptotic effect of rhIGF-1 (Liu *et al*, 1997) was not observed (Supplementary Fig S1D).

### rhIGF-1 specifically stimulates proliferation of Treg but not other T-cell subtypes

A direct link between IGF-1 signaling and Treg activation *in vitro* was supported by the abrogation of rhIGF-1-mediated Treg proliferation through IGF-1R inhibition (Fig 2C) and by the Foxp3 mRNA induction after rhIGF-1 stimulation (Fig 2D). rhIGF-1 treatment affected characteristic surface markers of Treg cell activation linked with proliferation (Fisson *et al*, 2003), modestly but significantly upregulating CD71 (Supplementary Figs S1E and S2A) and the activation marker CD44, and repressing the homing receptor CD62L (Fig 2E and Supplementary Fig S2A).

Treg proliferation (Fig 2F and Supplementary Fig S2B) and cell surface marker changes (Supplementary Fig S2A) were dependent on the activation of a canonical signalling pathway involving the PI3-kinase–Akt axis (Smith, 2010). Notably, rhIGF-1 had no stimulatory effect on CD4^+^CD25^−^ cells (Th0), or on the *in vitro* polarized IL-17 (Th17) and IFN-γ-secreting (Th1) pro-inflammatory subsets (Fig 2G), underscoring its potential to alter the balance of regulatory/pro-inflammatory cell subsets leading to a more immunosuppressive environment. Taken together, these data support a specific role for IGF-1 in positively regulating Treg cell-mediated immunosuppression.

### rhIGF-1 induces novel gene expression patterns associated with proliferation in Treg cells

**Figure 3:**
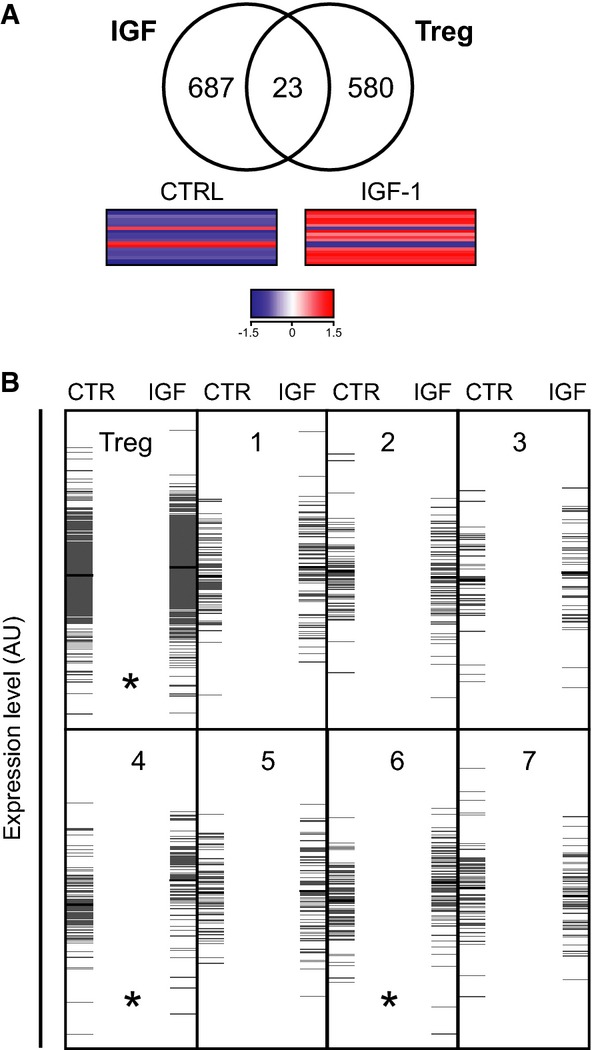
rhIGF-1 modulates the Treg cell transcriptional landscape Microarray analysis of 23 genes differentially expressed (> 2X) in Treg cells upon rhIGF-I stimulation (IGF) that overlap with the Treg signature (Treg) as defined by Hill *et al* (2007). Lower panel corresponds to a heatmap showing how this gene set is largely upregulated upon IGF-I stimulation (*P* = 0.025).rhIGF-1 treatment affects the expression of Treg signature genes (upper left subpanel; *P* = 0.004) and different functional clusters (as defined by Hill *et al*, 2007). Of the different clusters defined by Hill *et al*, only genes belonging to Cluster 4 (directly responsive to TCR and IL-2 cues; *P* = 0.0004) and Cluster 6 (not influenced by activation, TGF-β signaling or FoxP3; *P* = 0.001) are significantly upregulated by rhIGF-I treatment. Each gray horizontal line represents a gene expression level along an arbitrary unit (AU) scale on the *y*-axis. Average expression for each lane within each cluster is shown as a horizontal black line. The asterisk in subpanels “Treg” and Clusters 4 and 6 indicates significant differential expression between CTR (control) and IGF (rhIGF-I) treatment. *P < 0.05.

To further characterize the effects of IGF-1 on the Treg cell subset, we compared gene expression profiling of FACS-sorted mouse FoxP3^+^ cells to those stimulated with rhIGF-1 (Supplementary Table S1), which upregulated the majority of the overlapping 23 Treg cell signature genes (Hill *et al*, 2007) (Fig 3A). Of the seven canonical clusters of highly co-regulated genes within the Treg cell signature (Hill *et al*, 2007), two clusters (4 and 6, Fig 3B) were significantly affected by rhIGF-1 treatment. Cluster 4 includes genes directly responsive to TCR and IL-2 cues, consistent with the induction of Treg cell proliferation. Cluster 6, however, includes orphan genes not influenced by cell activation, TGF-β or FoxP3 (Supplementary Table S2). Transcription target enrichment analysis of Cluster 6 identified different gene sets associated with E2F transcription factors (Supplementary Table S3) involved in the regulation of cell cycle and proliferation (Johnson *et al*, 1993). Taken together, these results confirm that IGF-1 modulates a novel expression pattern of genes involved in Treg cell proliferation.

### Systemic delivery of IGF-1 protects from drug-induced and genetic diabetes

Type-1 diabetes (T1D) is an autoimmune disease caused by T-cell-induced destruction of the insulin-producing β-cells of the pancreas (Atkinson & Leiter, 1999). Disruption of the homeostatic balance of autoaggressive cells and Treg cells promotes T1D (Waid *et al*, 2008). To test the therapeutic potential of systemic rhIGF-1 delivery in T1D, wild-type C57/Bl6J female mice were implanted with a subcutaneous osmotic minipump containing rhIGF-1 and diabetes was induced by multiple low-dose streptozotocin (STZ) injections (Like & Rosini, 1976; Rossini *et al*, 1978; Paik *et al*, 1980; Nakamura *et al*, 1984; Linn *et al*, 1987; Cockfield *et al*, 1989; Yanagawa *et al*, 1989; Papaccio *et al*, 1994; Lukić *et al*, 1998; Stosić-Grujicić *et al*, 2009; Wen *et al*, 2009; Zdravkovic *et al*, 2009). Minipumps provided steady elevated systemic rhIGF-1 levels over 3 weeks (Supplementary Fig S2C). No adverse reaction to the human IGF-1 protein was seen, presumably due to the high sequence conservation with the mouse.

**Figure 4:**
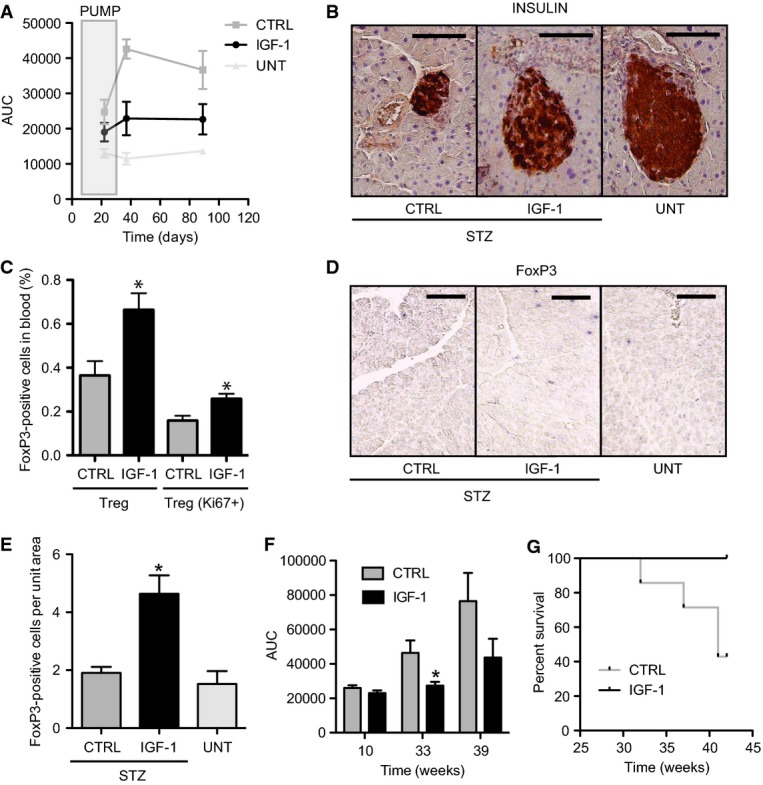
Systemic delivery of IGF-I protects from low-dose STZ-induced and spontaneous diabetes and increases pancreatic Treg cell numbers A Glucose tolerance tests (GTT) were performed at the indicated times from the first STZ injection, and the area under the curve (AUC) was calculated. rhIGF-1 delivery during 28 days (gray box; PUMP) improved glucose homeostasis (*P*_22d_ = 0.143, *P*_37d_ = 0.016, *P*_89d_ = 0.036; *n* = 13). B Insulin staining of day 97 pancreas reveals long-lasting protective effects of rhIGF-1 treatment on pancreatic cell mass and architecture in rhIGF-1-treated mice (IGF-1) compared to STZ-treated (CTRL) and untreated (UNT) mice. Bar = 0.1 mm. C Flow cytometric analysis of peripheral blood 1 week after implantation of rhIGF-1 minipumps shows increased FoxP3^+^ (Treg; *P* = 0.011, *n* = 13) and KI67^+^ (Treg Ki67; *P* = 0.007) Treg cells in STZ-treated animals. *P < 0.05. D, E Immunohistochemical analysis showing increased infiltration of FoxP3^+^ cells in pancreatic tissue of rhIGF-1-treated mice (*P = 0.015; *n* = 12) compared to STZ-treated (CTRL) and untreated (UNT) mice. Bar corresponds to 0.1 mm. F Glucose tolerance tests (GTT) were performed at the indicated times, and the area under the curve was calculated (AUC). Systemic rhIGF-1 delivery during 28 days (week 9–12) ameliorates diabetes progression in NOD mice (IGF-1; *P*_33w_ = 0.007, *P*_39w_ = 0.053; *n* = 14) compared to control mice (CTRL). *P < 0.05. G Systemic rhIGF-1 delivery increases NOD survival rate (*P* = 0.015; *n* = 14).

STZ treatment induced the first signs of deregulated glucose homeostasis after 3 weeks (Fig 4A and Supplementary Fig S2D). Similar to the effects previously observed in diabetic patients (LeRoith & Yakar, 2007), systemic delivery of rhIGF-1 improved glycemic control in these animals (Fig 4A and Supplementary Fig S2D). Notably, rhIGF-1 administration resulted in improved glucose homeostasis well beyond its direct hypoglycemic effects (LeRoith & Yakar, 2007) (Fig 4A), providing long-lasting protection of the cell mass and architecture of the glucose-responsive insulin-producing pancreatic islands (Fig 4B and Supplementary Fig S2E). Treatment with rhIGF-1 had no effect on glucose control in non-diabetic controls (Supplementary Fig S2F).

Since the homeostatic balance of Treg cells and auto-aggressive cells is critical in the development of autoimmune diabetes (Waid *et al*, 2008; Feuerer *et al*, 2009; Bluestone *et al*, 2010), we examined Treg cell numbers in peripheral blood and spleen. Measuring FoxP3 expression, we confirmed an increase in the absolute Treg cell numbers and those relative to CD4 after subcutaneous implantation of rhIGF-1 minipumps (Fig 4C and Supplementary Fig S2G–J), while CD4^+^ cells remain unchanged or decreased in peripheral blood or spleen, respectively (Supplementary Fig S2K and L). Increased Treg cells were also observed in the pancreata of STZ diabetic mice treated with rhIGF-1, as revealed by immunostaining against FoxP3 in diabetic or untreated mice (Fig 4D and E). Thus, relatively short but constant rhIGF-I treatment prevents the development and progression of disease by increasing peripheral Treg cell numbers and stably recruiting them to damaged pancreatic tissue, preventing islet degeneration.

As the STZ model of T1D is caused by direct toxicity to pancreatic β-cells (Lenzen, 2008), we also tested an alternative, genetic model of T1D, the non-obese diabetic (NOD) mouse, which spontaneously develops autoimmune diabetes at 10 weeks (Anderson & Bluestone, 2005). NOD mice were implanted with a subcutaneous osmotic minipump containing rhIGF-1 before diabetes onset (from week 9 to 12). As previously shown by daily subcutaneous injections of rhIGF-1 to prediabetic NOD mice (Bergerot *et al*, 1995; Kaino *et al*, 1996), we observed a delayed onset of diabetes symptoms. rhIGF-1 treatment not only improved glucose homeostasis (Fig 4F and Supplementary Fig S2M) but also reduced mortality (Fig 4G). Thus, continuous systemic delivery of rhIGF-1 afforded protection from both environmentally induced and spontaneous T1D.

### EAE amelioration after rhIGF-1 treatment is dependent on Treg cell function

**Figure 5:**
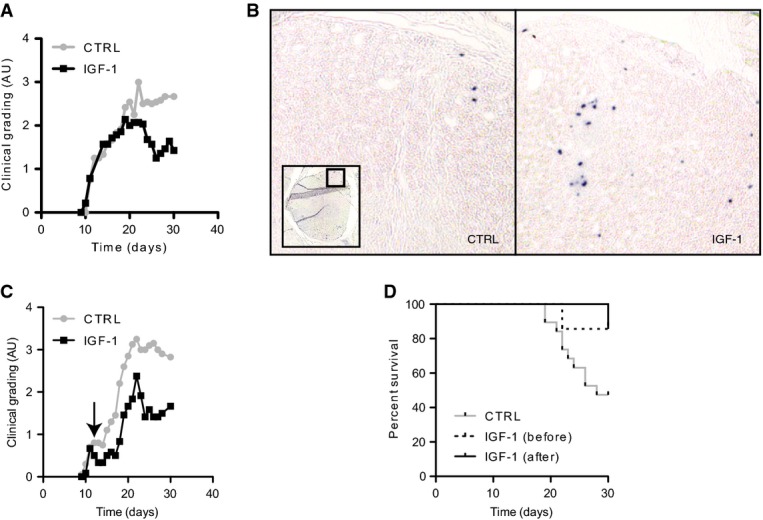
EAE amelioration after rhIGF-1 treatment is dependent on Treg cell function Clinical grading (1: no clinical signs; 4: forelimbs paralyzed) determined over 4 weeks after rhIGF-1 treatment, initiated 3 days before immunization with MOG_35–55_ peptide (day 0) (*P* = 0.01; *n* = 13).Mice treated with MOG_35–55_ peptide^+/−^ rhIGF-1 as in (A) showed increased FoxP3 infiltrating cells in the spinal cord induced by rhIGF-1 treatment (*P* = 0.014; *n* = 15).rhIGF-1 minipumps implanted after EAE induction when the first signs of disease appeared (day 11, see arrow) reduced clinical severity by 58% percent (e.g. day 26; *P* = 0.0296; *n* = 18).rhIGF-1 increased survival rates (*n* = 34, *P* = 0.028, log-rank test for trend) when administered before or after the induction of EAE.

To establish a broader role for IGF-1 in restoring immune tolerance, we exploited a murine model of multiple sclerosis, a chronic autoimmune demyelinating disease of unknown etiology. After subcutaneous implantation of rhIGF-1 minipumps, experimental autoimmune encephalomyelitis (EAE) was induced in mice with peptide MOG_35-55_ immunogen as described (see Materials and Methods). rhIGF-1 improved the clinical outcome of the disease, with consistently beneficial effects after the third week post-disease induction (Fig 5A). Treg cell numbers were increased in the spinal cords of rhIGF-1-treated animals at the early stages of the disease (Fig 5B and C). Both clinical improvement and increased Treg cell numbers associated with rhIGF-1 treatment soon after disease onset were abolished by CTLA-4 blockade (Supplementary Fig S3A–C), which interferes with Treg cell function (Herman *et al*, 2004), suggesting that the effects of rhIGF-1 on the T regulatory subset are required for the amelioration of the disease.

The beneficial action of rhIGF-1 extended to a more therapeutically relevant intervention when delivered after appearance of the first signs of EAE-induced paralysis. Reduction in clinical severity was observed soon after the initiation of rhIGF-1 treatment at day 11 post-MOG_35–55_ immunization (Fig 5E), which was as effective in reducing mortality as when administered prophylactically (Fig 5F). As in the diabetic model, rhIGF-1 increased the number of Treg cells in the affected tissue and improved clinical outcome.

### IGF-1/IGF-1 receptor axis directly regulates Treg cell proliferation and function *in vivo*

**Figure 6:**
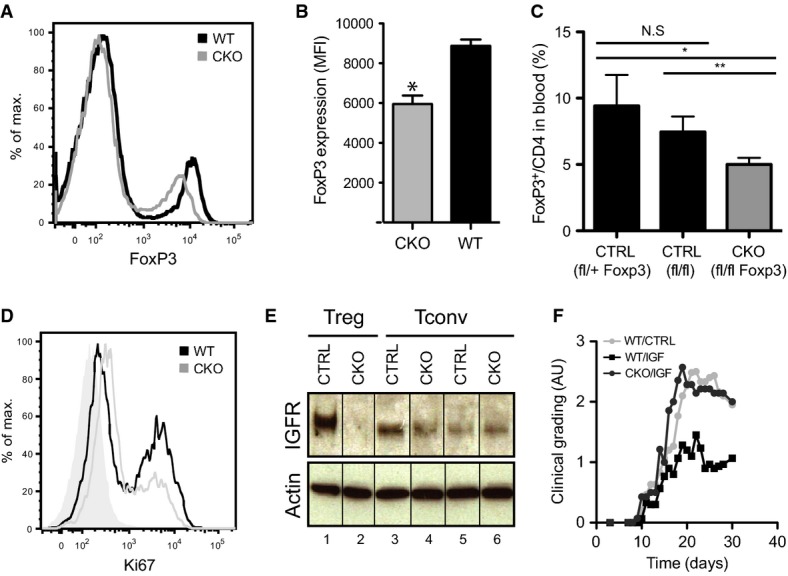
IGF-1/IGF-1 receptor axis regulates the proliferation and function of Treg cells *in vivo* A, B IGF-1 receptor-deficient Treg cells (*Foxp3*^*cre*^ *Igf1r*^*fl/fl*^) express lower levels of FoxP3 (mean fluorescent intensity; *P < 0.001, *n* = 16) upon contact hypersensitivity challenge. C Flow cytometric analysis of *Foxp3*^*cre*^ *Igf1r*^*fl/fl*^ (KO), *Igf1r*^*fl/+*^ and *Igf1r*^*fl/fl*^ (CTRL) peripheral blood CD4^+^ cells showing decreased FoxP3^+^ Treg cells in contact-hypersensitized *Foxp3*^*cre*^ *Igf1r*^*fl/fl*^ mice (*n* = 26; *P = 0.0049; **P = 0.021). D Representative flow cytometric analysis of splenic FoxP3^+^ cells shows decreased proliferation (Ki67 expression) in Treg cells of contact-hypersensitized *Foxp3*^*cre*^ *Igf1r*^*fl/fl*^ mice. Tinted plot corresponds to isotype control. E Top panel: IGF-1 receptor Western blot analysis of different populations of CD4-positive cells from *Igf1r*^*fl/fl*^ (CTRL) and *Foxp3*^*cre*^ *Igf1r*^*fl/fl*^ (KO) mice showing specific deletion of the IGF-1 receptor in the CKO Treg population. Lanes 1, 2: Treg cells (CD4^+^CD25^high^), Lanes 3, 5: memory CD4^+^CD25^−^CD44^+^CD62L^−^ T cells; Lanes 4, 6: naive CD4^+^CD25^−^CD44^−^CD62^+^ T cells. Bottom panel: Loading controls (β-actin). F rhIGF-1 minipumps were implanted after EAE induction when the first signs of disease appeared as in Fig 5E (day 11) and clinical grading was determined over a period of 4 weeks (2-way ANOVA, *P* = 0.048, *n* = 36).

The effects of rhIGF-1 described above could be explained by its direct stimulatory action on proliferation of the Treg subset, on increased Treg cell activation and migration to the damaged tissue, or a combination of both. A direct role for IGF-1 on Treg cell proliferation was assigned by analyzing mice carrying a Treg cell-specific deletion of the IGF-1 receptor (designated CKO). CKO mice were generated by crossing mice carrying a Foxp3 EGFP/Cre fusion cassette (Zhou *et al*, 2009) with an Exon3-floxed allele of the IGF-1R gene (*Foxp3*^*cre*^*Igf1r*^*fl/fl*^ (Temmerman *et al*, 2010) (Supplementary Fig S3D). CKO mice were indistinguishable from *Foxp3*^*cre*^ and *Igf1r*^*fl/fl*^ controls (CTRL) (Supplementary Fig S3E–I) until subjected to immune challenge (contact hypersensitivity, CHS), whereupon Treg cells showed reduced FoxP3 expression levels (Fig 6A and B), as well as a decrease in cell number and proliferative status (Fig 6C, D and Supplementary Figs S3J, K, and S4A–C). No phenotypic differences were noted between CTRL genotypes (Supplementary Tables S4 and S5).

Western blot analysis of IGF-1 receptor levels in CD4^+^ cell populations (Treg and T conventional (Tconv) cells, CD4^+^CD25^−^CD44^+^ and CD4^+^CD25^−^CD62L^+^) from CTRL and CKO mice showed depletion of the IGF-1 receptor specifically in the CD4^+^CD25^+^ Treg containing population (Fig 6E). T-cell activation also induced IGF-1 receptor expression in CTRL Tconv cells, albeit at lower levels in the CKO background. This was presumably due to indirect feedback from Treg CKO cells, as no *Foxp3*^*Cre*^ (GFP) expression was evident in these cells (Supplementary Fig S4D and E).

When tested in the EAE model, the collective therapeutic effect of rhIGF-1 was completely abolished in the CKO background (Fig 6F) establishing a direct link between blockade of disease progression and IGF-1-mediated Treg cell activation. Together, these data demonstrate that IGF-1 directly and specifically stimulates the proliferation of Treg cells *in vivo*, and provide a mechanism whereby IGF-I can modulate the quality and the amplitude of the immune response to inflammatory insults.

## Discussion

In this study, we report on the direct activation of human and mouse Treg cell proliferation by rhIGF-1 and demonstrate how a confined period of continuous systemic rhIGF-1 delivery using a clinically relevant, accepted method for delivering drugs in humans (Yaturu, 2013) is a feasible and readily applicable therapeutic avenue for the treatment of autoimmune and inflammatory diseases. Although recombinant IGF-1 is already in widespread use for the treatment of short stature in children (Richmond & Rogol, 2008), results in treating chronic disease have been mixed. Early studies of systemic IGF-1 delivery in diabetic patients led to decreased glucose and insulin levels during the time of administration, improving glycemic control (Zenobi *et al*, 1992; Carroll *et al*, 1998; LeRoith & Yakar, 2007). In a large study, diabetic subjects receiving IGF-I for 12 weeks experienced major decreases in insulin requirements (Morrow *et al*, 1994), but side effects included edema, jaw pain, tachycardia and worsening of retinopathy (Quattrin *et al*, 1997).

The effects of IGF-1 have also been tested in different models for multiple sclerosis. Initial reports (Yao *et al*, 1995; Liu *et al*, 1997 and references therein) assumed a direct effect of IGF-1 on oligodendrocytes and myelin regeneration, resulting in clinical amelioration. However, in subsequent studies, Lovett-Racke *et al* (1998) failed to observe any protection when IGF-1 was administered after disease onset or during the chronic phase of the disease (Cannella *et al*, 2000). Local IGF-1 administration also showed no effect on remyelination of aged mice (O'Leary *et al*, 2002) or on protection of mice from EAE (Genoud *et al*, 2005). Collectively, these studies indicated that IGF-1 therapy provided little protection and could even be deleterious if delivered together with IGFBP3 after disease onset (Lovett-Racke *et al*, 1998).

In contrast to these reports, we observed significant and lasting improvements in animal models of immune disease treated with a relatively short but uninterrupted supply of systemic rhIGF-1. The beneficial effects of rhIGF-1 on glucose homeostasis in diabetic mice extended far beyond the treatment itself, indicating durable improvement in pancreatic function. In the EAE model, continuous rhIGF-1 delivery produced clear amelioration even after the antigen response was triggered, presumably due to blockade of autoimmune progression and re-establishment of tolerance.

Despite extensive study, the precise mechanism of IGF-1 action in countering autoimmune disease has remained obscure. Previous reports implicating IGF-1 in the prevention of T1D development (Bergerot *et al*, 1995; Kaino *et al*, 1996; George *et al*, 2002; Chen *et al*, 2004; Casellas *et al*, 2006; Agudo *et al*, 2008) have cited its mitogenic properties (Smith *et al*, 1991; Le Roith, 1997) mainly acting on the affected tissues, while protecting them from the stress caused by the autoimmune attack. By establishing Treg cells as a critical contributor to the therapeutic effects of systemic IGF-1 delivery in autoimmune disease, our report builds on previous studies documenting decreased Treg numbers and FoxP3 expression in the inflamed pancreas of autoimmune mice (Feuerer *et al*, 2009; Bluestone *et al*, 2010). Although a recent study found intrapancreatic Treg cell numbers were increased by IGF-1 overexpression in the liver of STZ-treated mice (Anguela *et al*, 2013), an indirect effect was proposed, mediated by IL-7-producing dendritic cells that improved Treg survival or by the conversion of conventional T cells into Treg cells by TGF-β secreted from the liver. In contrast, the current study shows that the protective action of exogenous rhIGF-1 against autoimmune insults can be attributed to the direct stimulation of Treg cell proliferation, a key finding given that continuous self-renewal is the major mechanism of maintenance of this lineage in adulthood (Rubtsov *et al*, 2010).

Using a continuous delivery protocol, we show that rhIGF-1 not only stimulates proliferation of Treg cells but also alters their trafficking, allowing for homing in the damaged organ. Although significant numbers of Treg cells localize to the spinal cord during EAE (Korn *et al*, 2007; this work), their activity appears insufficient to prevent the autoimmune response. Webster *et al* (2009) showed that a well-defined Treg cell-stimulating cytokine (IL-2) led to a widespread expansion of this subset and to disease protection. However, this effect was only observed when IL-2 treatment preceded disease onset. However, unlike IGF-1, IL-2 treatment failed to induce Treg recruitment to the injured tissue. The inability of the expanded Treg cells to home in the damaged tissue might also lie behind the failure of an IL-2-based therapy for diabetes (Long *et al*, 2013); indeed, supplementation with IL-2 further augmented the proliferative effects of rhIGF-1 on Tregs *in vitro*. Consistent with the report of a CD44^hi^ Treg subset with high proliferative activity *in vivo* (Min *et al*, 2007)*,* we show here that Treg cells treated with rhIGF-1 acquire an activated memory-like phenotype (CD44^hi^, CD62L^lo^; Feuerer *et al*, 2009; Campbell & Koch, 2011), allowing them to effectively migrate to areas of inflammatory activity rather than to secondary lymphoid organs (Fisson *et al*, 2003; Bromley *et al*, 2008). Thus, continuous rhIGF-1 delivery is an attractive option for the therapeutic treatment of even advanced autoimmune patients.

Although a number of autoimmune diseases have been superficially examined for their potential association with abnormalities in the IGF-I/IGF-IR pathway (Smith, 2010), a substantial link between this pathway and the pathogenesis of these diseases has not been established. What is the precise role played by IGF-1/IGF-1R signaling in Treg cells? Continuous expression of FoxP3 is needed to maintain lineage identity and function of mature Treg cells (Williams & Rudensky, 2007), such that attenuation or loss of FoxP3 expression leads to defective Treg suppressive function and conversion into effector cells, enhancing rather than inhibiting the immune disease state (Wan & Flavell, 2007; Zhou *et al*, 2009). We therefore surmise that the therapeutic effects of rhIGF-1 delivery occur through direct stimulation of IGF-1R signaling, which stabilizes the Treg transcriptional landscape and enhances FoxP3 expression.

Of note in this respect is the ability of IGF-1 to specifically affect two Treg signature clusters of co-regulated genes (Clusters 4 and 6) not directly controlled by FoxP3 (Hill *et al*, 2007). Whereas genes in Cluster 4 are related to proliferation and activation, Cluster 6 contains genes for which IGF-1 seems to be a new regulator. Among the genes belonging to this latter cluster is the IGF-1 receptor itself (Hill *et al*, 2007), which stimulates proliferation and maintenance of Treg cells *in vivo* (Rubtsov *et al*, 2010).

By establishing a direct link between IGF-1 signaling, Treg cell activation and the re-establishment of the immune balance, we provide a rational for reinterpreting the findings and failures of past approaches (e.g. expressing IGF-1 in cells of the CNS in EAE models), protocols (e.g. short-term delivery of IGF-1 with only transient beneficial effects) and delivery methods (continuous delivery by pumping device versus subcutaneous injections). Together with a parallel study, in which allergic contact dermatitis was suppr-essed through IGF-1-mediated Treg cell activation (Johannesson *et al*, 2014), our results provide a viable protocol for stimulat-ing human Treg cell proliferation *in vitro* and *in vivo* and will also help in the search for appropriate clinical and surrogate markers for Treg cell expansion to aid in successful experimental and clinical design of autoimmune therapies. Given recent evidence of Treg involvement in muscle repair (Burzyn *et al*, 2013), it is likely that local stimulation of Treg cell expansion by IGF-1 may contribute to its well-documented beneficial effects in tissue regeneration as well.

## Materials and Methods

### *In vivo* experiments

All mice used were on a C57BL/6J genetic background unless otherwise indicated. *Igf1r*^*fl/fl*^*Foxp3*^*cre*^ mice were generated by crossing *Igf1r*^*fl/fl*^ (C57BL/6J; Jackson Laboratory; Temmerman *et al*, 2010) and *Foxp3*^*cre*^ (NOD; Jackson Laboratory; Efstratiadis *et al*, 2008) mice. rhIGF-1 (Biovision) was delivered continuously (0.25 μl/h, 28-day release) via subcutaneously implanted Alzet osmotic minipump (model #2004, Alzet Osmotic Pumps Company) at a dose of 0.275 mg/kg/day. Surgical implantation of the osmotic minipumps was performed 1 week before the induction of diabetes or EAE, unless otherwise indicated. Control mice were either sham-operated mice or implanted with PBS (solvent)-delivering pumps, as no difference was observed between these two groups compared to rhIGF-1-delivering pumps (Supplementary Fig S4F). rhIGF-I levels were determined by ELISA (Human IGF-I Quantikine, R&D) according to the manufacturer's protocol.

Mice were maintained at EMBL Monterotondo Laboratory Animal Facility. All mouse procedures were approved by the EMBL Monterotondo Ethical Committee (Monterotondo, Italy) and were in accordance with national and European regulations. All animal colonies are housed in accordance with the European Legal framework that exists for the protection of animals used for experimental and other scientific purposes (European convention ETS123/Council of Europe, European directive 86/609/EEC and the recently published Directive2010/63/EU) as well as the current Guidelines of International Organizations such as the Association for the Assessment and Accreditation of Laboratory Animal Care International (AAALAC) and the Federation of European Laboratory Animal Science Association (FELASA). The macroclimate of the facility is controlled via a central monitoring unit; thus, the conditions of temperature, humidity, ventilation, air changes and dark/light cycles are under strict limits. Animals are hosted under specific pathogen-free (SPF) conditions in individual ventilated cages (IVC) and isolators. The animal facilities are subjected to a complete veterinary medical care program by the responsible veterinarian and manager (LAS specialist FELASA cat.D) which includes preventive medicine, surveillance, diagnosis, treatment and control of diseases as well as veterinary care of the animals used in experimental protocols. A health-monitoring program is also in force, in accordance with the guidelines issued by FELASA. The surveillance system personnel are also responsible for determining the humane end points and for deciding whether the animals should be euthanized in order to avoid further suffering. The 3Rs principle is routinely implemented in our experimental design in order to minimize the use of animals. Special attention is taken to minimize the number of animals included in each study, and experiments are designed to obtain maximum amount of data from each experimental mouse.

Human data were obtained under informed consent and using procedures and protocols approved by the EMBL Bioethics Internal Advisory Committee (BIAC) and conforming to the principles set out in the WMA Declaration of Helsinki and the NIH Belmont Report.

Diabetes experiments were performed using NOD mice (Jackson Laboratory), or C57BL/6J mice as described in the Low-Dose Streptozotocin Induction Protocol (Diabetic Complications Consortium). (http://www.diacomp.org/shared/showFile.aspx?doctypeid=3&docid=19). Briefly, freshly prepared streptozotocin (Sigma) solution was injected intraperitoneally at a dose of 40 mg/kg. Mice received one injection each day for 5 days consecutively. Glucose homeostasis was determined at the indicated time points using an intraperitoneal glucose tolerance test (Heikkinen *et al*, 2007). After having measured basal blood glucose concentrations, a 20% aqueous glucose solution was administered intraperitoneally to fasted mice at a dose of 2 g glucose/kg. Clearance of glucose in blood was measured at 30-min intervals for a period of 3 h. For statistical analysis, the area under the curve was calculated using Prism (GraphPad Software).

EAE experiments were performed as described (Stromnes & Goverman, 2006) using the peptide MOG_35–55_ as an immunogen. Briefly, mice were injected with a 1:1 emulsion of the MOG_35–55_ peptide (200 μg/mouse; AnaSpec) and complete Freund's adjuvant (Sigma) at day 0. Two doses of pertussis toxin (400 ng/mouse; Sigma) were administered at day 0 and day 2. Mice were then monitored for clinical signs and weight loss. Clinical severity was scored using a grading scale 0–5 where the following grades correspond to the indicated clinical signs (0, No clinical signs; 0.5, Partially limp tail; 1, Paralyzed tail; 2, Loss in coordinated movement, hind limb paresis; 2.5, One hind limb paralyzed; 3, Both hind limbs paralyzed; 3.5, Hind limbs paralyzed, weakness in forelimbs; 4, Forelimbs paralyzed; 5, Moribund). In the experiment recreating therapeutic intervention with *Igf1r*^*fl/fl*^*Foxp3*^*cre*^ mice, rhIGF-1 minipumps were implanted after EAE induction when the first signs of disease appeared and clinical grading was determined over a period of 4 weeks, and only in mice showing symptoms of paralysis for at least two consecutive days. Where indicated, mice were injected at day 0 with purified anti-CTLA-4 (UC10.4F10.11, BD Pharmingen) antibody or isotype control (A95-1; BD Horizon) at a dose of 0.6 mg/mouse.

Direct proliferative stimulation of Treg cells by IGF-I *in vivo* was determined by flow cytometric analysis of CD4-positive cells from *Igf1r*^*fl/fl*^*Foxp3*^*cre*^ spleens in the context of a contact hypersensitivity response (Klekotka *et al*, 2010). Briefly, mice were sensitized on the abdomen with 50 μl of 0.5% DNFB (Sigma-Aldrich, Saint Louis, MO) on day 0 and day 1. Flow cytometric analysis of the spleens was performed on day 3 after elicitation. After tissue disaggregation, single cell suspensions were blocked with anti-CD16/32 and further stained with anti-CD4, FoxP3 and Ki67 antibodies according to the manufacturer's protocol.

### Histological analysis

Tissues were fixed in 4% formaldehyde overnight, dehydrated in an increasing gradient of ethanol and embedded in paraffin. In EAE experiments, parts of the vertebral column containing the spinal cord were fixed, decalcified in 0.5% EDTA for a week prior to embedding. Ten-micrometer sections were made and then dewaxed and rehydrated in an ethanol gradient. For FoxP3 and CD4 stainings, heat antigen retrieval was performed and endogenous peroxidase was quenched by a 10-min incubation in 1% H_2_O_2_. After blocking with 2% NGS, immunostaining was performed using a FoxP3 antibody (Abcam) and a secondary anti-rabbit-AP (Sigma), CD4 (Abcam) or proinsulin (Abcam) and Vectastain ABC kit according to the manufacturer's protocol. DAB solution (Sigma) was used for signal detection with HRP-conjugated antibodies or NBT/BCIP (Roche) with AP-conjugated antibodies. Proinsulin-stained sections were counterstained with hematoxylin.

Images were captured using a LMD7000 (Leica) microscope and quantified manually using ImageJ cell counter. At least three sections from each mouse separated from each other by 50 μm were analyzed.

### *In vitro* experiments

Antibodies against CD16/32 (Clon 93), CD4 (GK1.5), CD25 (PC61.5), FoxP3 (FJK-16F), IFN-γ (XMG1.2), IL-17 (eBio17B7), CD71 (RI7 217.1.4) and CD44 (IM7) were purchased from eBioscience. Anti-CD62L (MEL-14), anti-Ki-67 and annexin V were purchased from BD Pharmingen. For the experiment with human cells, antibodies were purchased from BD Pharmingen, including the regulatory T-cell cocktail, FoxP3 staining kit and buffer set. Surface and intracellular stainings were performed according to the manufacturer's protocol. For the Western blot analysis, a rabbit mAb IGF-I Receptor β (D23H3) XP® (Cell signaling Technology) was used.

For *in vitro* murine proliferation experiments, cells were isolated from spleens of C57BL/6 mice. After red blood lysis, cells were incubated sequentially with anti-CD16/32 and directly labeled anti-CD4 and CD25 antibodies. CD4 CD25 double-positive cells were then sorted with a standard three-laser FACS Aria or a 5-laser FACS Aria SORP from BD (70 μm nozzle, 70 psi; > 98% purity) and stimulated with coated anti-CD3 (17A2, eBioscience) and soluble anti-CD28 (37.51, eBioscience) and the indicated factors or inhibitors (IGF-1R inhibitor PPP, Calbiochem; Ly-294,002, Sigma; Deguelin, Sigma; PD.98,059, Sigma). IGF-1 was used at a concentration of 25–100 ng/ml unless otherwise indicated.

For the *in vitro* human proliferation experiment, mononuclear cells from EDTA-treated blood were isolated by Ficoll-hypaque (Pharmacia Biotech) gradient centrifugation and stained with the human T regulatory cocktail. Treg cells were then sorted using a 5-laser FACS Aria SORP from BD (85-μm nozzle, 40 psi; > 98% purity) and stimulated with anti-CD3/anti-CD28-coated beads (Gibco), and the indicated factors in RPMI supplemented with 10% FCS for a period of 6 days. After surface staining, cells were fixed and stained for FoxP3 and Ki67 and analyzed in a FACS Aria SORP.

For *in vitro* polarization experiments, FACS-purified CD4-positive CD25-negative cells were pre-incubated for 3–5 days with IFN-γ (PeproTech), IL-12 (PeproTech) and anti-IL-4 antibodies (11B11; eBioscience) (Th1) or TGF-β (PeproTech), IL-6 (PeproTech), anti-IL-4, anti-IL12 (C17.8; eBioscience) and IFN-γ (AN-18; eBioscience) antibodies (Th17). The number of FoxP3-positive cells was determined by flow cytometric analysis in a two-laser FACS Canto (BD Biosciences) or a FACS Aria (BD Biosciences) after intracellular staining according to the manufacturer's protocol (IC fixation buffer or FoxP3 staining buffer set, eBioscience). Data analysis was performed using FloJo (Tree Star Inc.) or FACS Diva software (BD Biosciences). When a representative example is shown, the experiment was repeated at least three times.

Suppression experiments were performed with sorted FACS-purified splenic cells. Briefly, sorted CD4-positive CD25-negative cells were first stained with carboxyfluorescein diacetate succinimidylester (CFSE) (Molecular Probes). Cells were then cultured for 3 days in 96-well plates with irradiated antigen-presenting splenocytes (30 Gy), soluble anti-CD3 (145.2C11; eBioscience) and the indicated ratios of CD4 CD25 double-positive cells previously treated with rhIGF-1. The percentages of proliferating and non-proliferating cells were then used to calculate the degree of suppression.

### Gene expression profiling

Treg cells were isolated from spleens of *Foxp3*^*cre/gfp*^ mice, purified by FACS (CD4^+^ CD25^+^ GFP^+^), incubated with rhIGF-1 for 2 days and further purified by FACS. RNA was prepared with the RNeasy Mini Kit according to the manufacturer's instructions (Qiagen). Two rounds of RNA amplification, labelling and hybridization to M430 2.0 chips (Affymetrix) were done at the EMBL Gene Core Facility in Heidelberg. Microarray data were analyzed using the GeneSpring software, and differential expression was defined by two-fold with a false discovery rate of *P* < 0.05. Microarray results were confirmed by quantitative RNA analysis performed by real-time PCR using Taqman probes (Applied Biosystems, Life Technologies) corresponding to a subset of genes (*stat1, bcl1 1b, map3k2, mapk1, tnfsf11, cd44, crebp* and *foxo1*). The mRNA amounts were normalized to those of the *hprt* gene. Transcription target enrichment analyses were performed using the web-based tool WebGestalt2 (Zhang *et al*, 2005; Duncan *et al*, 2010). The microarray data performed in this study were submitted to ArrayExpress database [http://www.ebi.ac.uk/arrayexpress/] and received the accession number E-MTAB-2951.

### Statistics

All analyses were performed with Prism 5.0 (GraphPad Software Inc.). Unless otherwise indicated, statistical analyses were performed by a nonparametric Mann–Whitney *U*-test or Student's *t*-test and graphs show the mean and standard error. Specific tests and significance levels are found in figure legends.
