# Supplementary material for: Insulin-like growth factor-1 stimulates regulatory T cells and suppresses autoimmune disease
Source: EMBO Mol Med. 2014 Nov 3;6(11):1423–35. doi: 10.15252/emmm.201303376 (PMC4237469; doi:10.15252/emmm.201303376)
Supplement: Supplementary file 1 [file emmm0006-1423-sd1.pdf]

EMBO
Molecular Medicine

Insulin-like growth factor-1 stimulates regulatory T cells and suppresses autoimmune disease

Daniel Bilbao, Luisa Luciani, Bjarki Johannesson, Agnieszka Piszczek & Nadia Rosenthal

Corresponding author: Daniel Bilbao, EMBL

Review timeline:

Submission date:	09 August 2013
Editorial Decision:	21 September 2013
Revision received:	11 February 2014
Editorial Decision:	07 March 2014
Revision received:	14 July 2014
Editorial Decision:	15 July 2014
Revision received:	23 September 2014
Accepted:	26 September 2014

Transaction Report:

(Note: With the exception of the correction of typographical or spelling errors that could be a source of ambiguity, letters and reports are not edited. The original formatting of letters and referee reports may not be reflected in this compilation.)

Editor: Céline Carret

1st Editorial Decision 21 September 2013

Thank you for the submission of your manuscript to EMBO Molecular Medicine. We have now heard back from the three referees whom we asked to evaluate your manuscript. Although the referees find the study to be of potential interest, they also raise a number of concerns that must be addressed in a major revision of the study.

Without repeating here their comments, I would like to emphasise that further experiments must be performed to address all issues raised in a convincing and conclusive manner. In addition, extension of the findings to include human cells would greatly increase the impact of the manuscript as mentioned by Referee 1. We realise that many experiments are suggested to strengthen the data, while they would require additional efforts and time, we feel that the study would be greatly improved and as such can only recommend you to perform these.

Should you be able to address all criticisms, we would be happy to consider a revised manuscript.

Please note that it is EMBO Molecular Medicine policy to allow a single round of revision in order to avoid the delayed publication of research findings. Consequently, acceptance or rejection of the manuscript will depend on the completeness of your responses included in the next version of the manuscript.

EMBO Molecular Medicine has a "scooping protection" policy, whereby similar findings that are published by others during review or revision are not a criterion for rejection.

Should you decide to submit a revised version, I do ask that you get in touch after three months if you have not completed it, to update us on the status.

Please also contact us as soon as possible if similar work is published elsewhere. If other work is published we may not be able to extend the revision period beyond three months.

I look forward to receiving your revised manuscript.

***** Reviewer's comments *****

Referee #1 (Comments on Novelty/Model System):

In their manuscript "Insulin-like growth factor-1 stimulates regulatory T cells and suppresses autoimmune disease", Bilbao et al. demonstrate that continuous infusion of human IGF1 can ameliorate development of T1 diabetes and multiple sclerosis in mice through its effects on regulatory T cells. Most interestingly, using conditional mutagenesis of the IGF1 receptor, they show that these effects are directly dependent on IGF1 receptor expression on T regs themselves. Many previous studies have evaluated the effects of IGF1 on autoimmunity, and a recent study implicated modulation of Treg numbers in this process (Anguela et al, Diabetes, 2013). Therefore, the major novel aspect of these studies is that they link the efficacy of IGF-1 treatment to its direct effects on Tregs. Although these studies are intriguing, additional controls appear to be necessary to solidify the authors' conclusions. Furthermore, extending these studies to show that IGF1 has similar effects on human Tregs in vitro would greatly increase the potential impact of these studies.

Major and minor criticisms:
As the findings using IGF1R conditional mice are some of the most important of this paper, it is essential to control for possible artifacts that often arise when using conditional knockouts. Firstly, it is unclear which controls were used in the experiments using IGF1Rfl/fl;Foxp3Cre mice. The authors should compare these mice with IGF1R+/+;Foxp3Cre mice and IGF1Rfl/fl mice in all critical experiments to be certain that their results are not artifacts. Second, the authors should directly show that IGF1Rfl/fl Foxp3 cre mice lack IGF1R specifically in Tregs. Comparison of IGF1R protein expression in conventional (resting and activated) T cells with expression in Tregs would be ideal.
The authors say on pg 9 that Treg IGF1KOs were normal until subjected to immune challenge, but do not show any data or elaborate on what was normal. These data should be included.
In Fig4C, the authors should include a KO control that has not received IGF-1 to test whether physiological IGF1 has any effect on Tregs during EAE.
The authors show that IGF1R-deficiency in Tregs prevents IGF1-mediated amelioration of EAE. It would be nice to test whether similar results are seen in Diabetes.
The authors show that IGF1R-deficiency prevents Treg expansion during contact hypersensitivity. Does skin painting for CHS induce IGF1 production?
The authors should extend their in vitro studies to include studies using human Tregs. This would greatly strengthen the potential impact of these studies.

Referee #1 (Remarks):

Overall, this is an interesting manuscript and may represent an important finding with respect to the mechanism by which IGF1 can ameliorate autoimmune disease. However, additional controls are necessary to make this manuscript appropriate for publication in EMBO Molecular Medicine. Furthermore, extension of these studies to include human cells would greatly increase the impact of this manuscript.

Referee #2 (Remarks):

In this study the authors suggest that IGF-1 stimulates regulatory T cells and suppresses EAE and

STZ injury induced model of diabetes.

While IGF-1 pump has an effect on the incidence of diabetes, a firm evidence that the observed effect is due to increase in Treg cell numbers is lacking.

There are also numerous additional issues with this study.

First, there are very few primary data shown. In all Figures, the data are heavily processed and meaningful conclusions cannot be drawn on their basis (see for example Fig. 1, Fig. 2, etc.). No single primary flow cytometric analysis is shown with the exception of Fig. 5A and E. The latter experiment, however, lacks statistical analyses.

The quality of histology data shown in Fig. 1 is insufficient to support the authors' conclusion.

The quality of suppression assay shown in Fig. 5B is impossible to judge in the absence of primary data.

In experiment shown in Fig. 3B control group does not have CTLA4 treated control group. Furthermore, treatment with anti-CTLA4 antibody is not a means of inducing depletion of Treg cells.

The data for EAE suggest that the disease is very weak at best and any decrease of a disease score 1 cannot be considered significant.

IGF1R knockout data cannot be interpreted as these mice have decreased expression for Foxp3 in Treg cells and fewer Treg cells and their immunological characteristics under physiologic conditions and under immune challenge have not been described.

In conclusion, this study is unsuitable for publication.

Referee #3 (Remarks):

This work suggests that IGF1 may preferentially promote Treg growth and function to control inflammation. The authors make use of Foxp3-cre mice to specifically delete IGF1R in Treg. Such mice are not responsive to IGF1-mediated suppression of EAE. In several instances, it is not possible to conclude that Treg are preferentially responding to IGF1. The following data should be included:
1. In the induced and spontaneous diabetes models (Fig 1 and 2), it is important to show the % Treg of CD4 T cells, including representative scatter plots. As the data is presented it is not possible if the increased Treg is due to an increase in all T cells.
2. The results in Fig 4 with the inducible deletion of IGF1R in Treg are more compelling; however, it is important to show the difference in MFI pre and post DTH challenge, and preferably with representative scatter plots in addition to the MFI bar graph. Here again (Fig 4b), the % Treg of CD4 T cells should be shown.
3. In Fig 5a, growth of purified Treg is usually performed with exogenous IL-2, as Treg do not survive well without it. Does exogenous IGF1 improve Treg growth in vitro when IL-2 is also included?
4. In Fig 5f, do polarized Treg express more IGF1R compared to the other 3 polarization cultures?

1st Revision - authors' response 11 February 2014

Referee #1 (Comments on Novelty/Model System): *In their manuscript "Insulin-like growth factor-1 stimulates regulatory T cells and suppresses autoimmune disease", Bilbao et al. demonstrate that*

continuous infusion of human IGF1 can ameliorate development of T1 diabetes and multiple sclerosis in mice through its effects on regulatory T cells. Most interestingly, using conditional mutagenesis of the IGF1 receptor, they show that these effects are directly dependent on IGF1 receptor expression on T regs themselves. Many previous studies have evaluated the effects of IGF1 on autoimmunity, and a recent study implicated modulation of Treg numbers in this process (Anguela et al, Diabetes, 2013). Therefore, the major novel aspect of these studies is that they link the efficacy of IGF-1 treatment to its direct effects on Tregs. Although these studies are intriguing, ***additional controls appear to be necessary to solidify the authors' conclusions****. Furthermore, extending these studies to show that* ***IGF1 has similar effects on human Tregs in vitro*** *would greatly increase the potential impact of these studies.*

(Remarks): *Overall, this is an interesting manuscript and may represent an important finding with respect to the mechanism by which IGF1 can ameliorate autoimmune disease. However,* ***additional controls*** *are necessary to make this manuscript appropriate for publication in EMBO Molecular Medicine. Furthermore, extension of these studies to include* ***human cells*** *would greatly increase the impact of this manuscript.*

We thank Reviewer #1 for constructive suggestions that have now been incorporated into the manuscript as outlined below.

Major and minor criticisms:

1. As the findings using IGF1R conditional mice are some of the most important of this paper, it is essential to control for possible artifacts that often arise when using conditional knockouts. Firstly, it is unclear ***which controls were used in the experiments using IGF1Rfl/fl;Foxp3Cre mice.*** *The authors should compare these mice with* ***IGF1R+/+;Foxp3Cre mice and IGF1Rfl/fl mice*** *in all critical experiments to be certain that their results are not artifacts.*

We have now included the requested controls in the IGF-1R CKO crosses (see Figs. S3A, B, C; Table S4, and Table S5). Reassuringly, there were no phenotypes observed in IGF1Rfl/+; IGF1Rfl/fl or Foxp3Cre mice, confirming that the results reported in the CKO (IGF1Rfl/fl;Foxp3Cre) mice were not artifactual.

2. Second, the authors should directly show that ***IGF1Rfl/fl Foxp3 cre mice lack IGF1R specifically in Tregs****. Comparison of* ***IGF1R protein expression in conventional (resting and activated) T cells with expression in Tregs*** *would be ideal.*

Fig. 6 now shows that $CD25^{high}$ cells (Treg population) lack IGF-1R, whereas $CD25^{-}$ cells, either $CD44^{+}$ or $CD62L^{+}$ retain the IGF-1R. As noted in Fig. 6D legend, $CD4^{+}$ $CD25^{-}$ $CD44^{+}$ cells in control mice expressed higher IGF-1R levels than those in CKO mice, presumably as an indirect consequence of IGF-1R depletion in the Treg population, since Foxp3Cre (visualized by co-expressed EGFP), was expressed only in the $CD25^{+}$ cells (Fig S3D and E) and enriched in the $CD25^{high}$ subset. Partial deletion of IGF-1R in the $CD4^{+}$ $CD25^{-}$ $CD44^{+}$, would result in a reduced population, whereas they were in fact increased in the CKO mice (data not shown). IGF-1R expression was unaffected in the $CD4^{+}$ $CD25^{-}$ $CD62L^{+}$ cells (Fig. 6D).

3. The authors say that Treg IGF1KOs were ***normal until subjected to immune challenge****, but do not show any data or elaborate on what was normal. These data should be included.*

Fig. S3A, B, C and Table S4 and S5 now include the requested data.

4. The authors should include a ***KO control that has not received IGF-1*** *to test whether physiological IGF-1 has any effect on Tregs during EAE.*

This control is included in the context of the CHS test (see Table S5). In summary, deletion of the IGF-1R had no effect on the phenotype either of the controls or of the CKO mice in an immune challenge such as CHS, indicating that physiological IGF-1 levels are insufficient to affect Treg cell activation/expansion.

5. The authors show that IGF1R-deficiency in Tregs prevents IGF1-mediated amelioration of EAE. It would be nice to test ***whether similar results are seen in Diabetes****.*

In the time allotted for resubmission, we could not recapitulate these crosses in the context of the two diabetes models, but rather have included a more extensive analysis of the diabetes model (see response to reviewer below).

6. The authors show that IGF1R-deficiency prevents Treg expansion during contact hypersensitivity. Does ***skin painting for CHS induce IGF1 production****?*

There is no evidence in the literature that DNFB application leads to IGF-1 production. Upon activation of the immune response through the CHS protocol, Treg cells likely receive a signal that makes them sensitive to exogenous IGF-1 and leads to their expansion. In the absence of the IGF-1 receptor, such activation/expansion is compromised.

7. The authors should extend their in vitro studies to ***include studies using human Tregs****. This would greatly strengthen the potential impact of these studies.*

These data are now provided in Figure 1.

<u>Referee #2 (Remarks):</u>

In this study the authors suggest that IGF-1 stimulates regulatory T cells and suppresses EAE and STZ injury induced model of diabetes. While IGF-1 pump has an effect on the incidence of diabetes, ***a firm evidence that the observed effect is due to increase in Treg cell numbers is lacking****. There are also numerous additional issues with this study.*

We thank Reviewer #2 for constructive suggestions but note that TWO models of diabetes were analyzed (STZ induced and NOD spontaneous).

1. *First, there are very few primary data shown. In all Figures, the data are heavily processed and meaningful conclusions cannot be drawn on their basis (see for example Fig. 1, Fig. 2, etc.). No single primary flow cytometric analysis is shown with the exception of Fig. 5A and E. The latter experiment, however, lacks statistical analyses.*

All primary data has now been included, see for example Fig. 1, Fig. 6, Fig. S1, Fig. S2 and Fig. S3. Statistical analyses for the experiments shown in Fig. 2E (previously 5E) are now shown in Fig

S2A.

2. The quality of histology data shown in Fig. 1 is insufficient to support the authors' conclusion.

Our histological data is equivalent or superior to many other similar analyses in the literature. However we have provided additional histological examples in raw tiff form (see separate data file for Reviewers: SuppFigReviewer_CTRL/IGF/UNT) that support our original claims.

3. The quality of suppression assay shown in Fig. 5B is impossible to judge in the absence of primary data.

An example of these primary data is now included in Supplemental Figure S1C.

4. In experiment shown in Fig. 3B control group does not have CTLA4 treated control group. Furthermore, ***treatment with anti-CTLA4 antibody is not a means of inducing depletion of Treg cells****.*

We agree with the reviewer that treatment with anti-CTLA4 antibody is not a means of inducing depletion of Treg cells. In fact, as previously shown in other models (e.g., Read et al., 2000; Herman et al., 2004), similar experiments provide direct evidence that the immunosuppressive function of CD25+ Treg cells is dependent on CTLA-4. Although the exact mechanism of CTLA-4 action in vivo is not known, it is expressed predominately on Treg cells. Our data suggest the effect of anti–CTLA-4 on Treg cells inhibits their function, as similar numbers of CD25+CD4+ cells were present in anti–CTLA-4–treated and untreated mice. Inhibiting Treg function might, however, lead to decreased proliferation or a failure in the recruitment to the spinal cord in the EAE model, as our data suggest. As CTLA-4 is also expressed as an inhibitory molecule on (activated) T effector cells, its function on Teff would also be blocked by in vivo CTLA-4 treatment, leading to higher Teff cell proliferation in response to challenge. Although not critical for interpretations of our central findings in vivo, the CTLA-4 data we have included are consistent with the rest of our results and represent an interesting and relevant observation that we would prefer to retain, however they are now presented in the Supporting information (Fig. S3A-C).

5. The data for EAE suggest that the disease is very weak at best and any decrease of a disease score 1 cannot be considered significant.

Counter to the reviewer's concerns, the average disease score in our EAE study reaches 3 (see Fig. 5A and Fig. 5E) and after day 20 some mice scored 5 (moribund or dead; see Fig. 5F), in accordance to published literature (e.g., Mangano et al., 2010).

If the reviewer is referring to the experiment in Fig. S3A-C (previously Fig. 3B), this is a consequence of the experimental set up of that particular experiment. Mice were injected with the antibody a week prior initiation of the EAE protocol. In this experiment, we sought an initial confirmation that Treg cell function was important for the IGF-1 protective effects that we had observed in other models. Bearing in mind that the antibody effects (due to clearance) have a limited span while development of the full disease can take several weeks, we focused on the early stages of the disease, where the disease scores could reveal a delay in the appearance of the first symptoms. As variability at these stages is high, it is noteworthy that even in those conditions, we scored a significant effect. Importantly, Treg cells were increased in the spinal cords even at these very early stages of the disease, supporting the hypothesis that IGF-1 helps expand Treg cells at the sites of inflammation.

*6. **IGF1R knockout data cannot be interpreted** as these mice have decreased expression for Foxp3 in Treg cells and fewer Treg cells and their immunological characteristics under physiologic conditions and under immune challenge have not been described.*

We now provide further characterization of these mice in Fig. S3, Fig. S4, Table S4 and Table S5.

However we do not fully understand the criticism of the reviewer. Foxp3 expression is associated with and essential for Treg cell function: in the context of wt Treg cells, stimulation with IGF-1 leads to increased Foxp3 expression *in vitro* (Fig. 2D), and activation/expansion. In the absence of that pathway (e.g., upon deletion of the IGF-1 receptor), basal expression of this transcription factor is maintained in homeostatic conditions (as shown in Fig. S3H, I). Upon immune challenge, wt Treg cells are stimulated (and have higher Foxp3 expression, Fig. 6A, B), while CKO Treg cells remain refractory to the IGF-1 stimulatory effect. We therefore conclude that the IGF-1/IGF-1 receptor axis is important for the activation/expansion of those cells under certain circumstances such as immune activation or autoimmunity.

*<u>Referee #3 (Remarks):</u> This work suggests that IGF1 may preferentially promote Treg growth and function to control inflammation. The authors make use of Foxp3-cre mice to specifically delete IGF1R in Treg. Such mice are not responsive to IGF1-mediated suppression of EAE. In several instances, **it is not possible to conclude that Treg are preferentially responding to IGF1**. The following data should be included:*

We thank Reviewer #3 for constructive suggestions, which have been incorporated as follows:

*1. In the induced and spontaneous diabetes models (Fig 1 and 2), it is important to show **the % Treg of CD4 T cells**, including representative scatter plots. As the data is presented it is not possible if the increased Treg is due to an increase in all T cells.*

These data have now been incorporated in Fig. S2 J-I, and S2K.

*2. The results in Fig 4 with the inducible deletion of IGF1R in Treg are more compelling; however, it is important to show the **difference in MFI pre and post DTH challenge**, and preferably with representative scatter plots in addition to the MFI bar graph.*

These data have now been incorporated in Fig. 6A and Fig. S3D and E.

*3. Here again (Fig 4b), **the % Treg of CD4 T cells** should be shown.*

These data have now been incorporated in Fig. S2F and S2J.

*4. In Fig 5a, growth of purified Treg is usually performed with exogenous IL-2, as Treg do not survive well without it. Does exogenous IGF1 improve Treg growth in vitro when **IL-2 is also included**?*

In preliminary experiments, we scored an additive effect of IL-2 and IGF-1 in vitro, and have provided these data for the Reviewer's consideration (see separate data file for Reviewers: SuppFigReviewer_IL2). However the potential interactions of IGF-1 and IL-2 warrant further investigation as the subject of a separate study to include the effects of IGF-1 with and without IL-2

in vivo, so we have elected not to include this single experiment, which is not the focus of the current study.

4. In Fig 5f, do ***polarized Treg express more IGF1R*** *compared to the other 3 polarization cultures?*

In all our experiments we used naturally occurring Treg cells. However, as now shown in Fig. 6D, naturally occurring Tregs do express more IGF-1R compared to other CD4 cells.

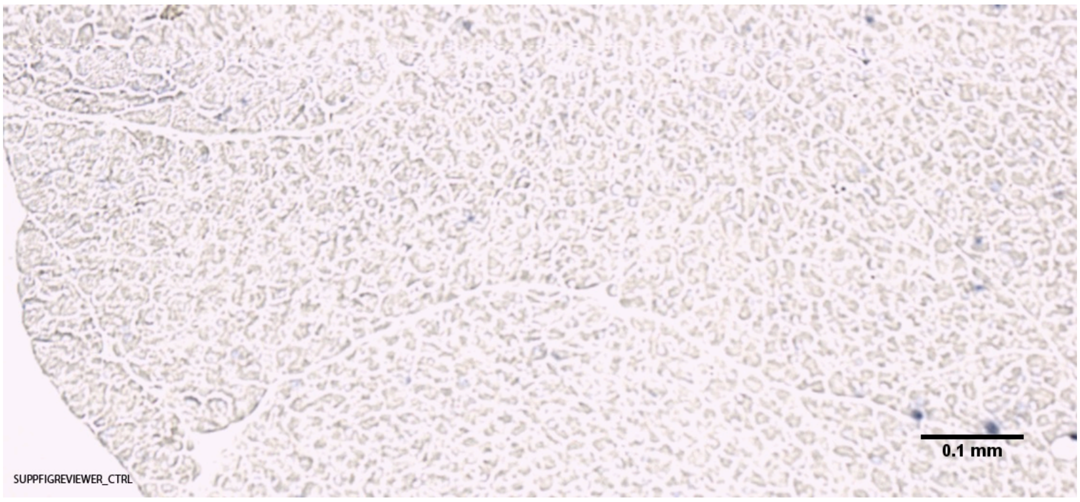

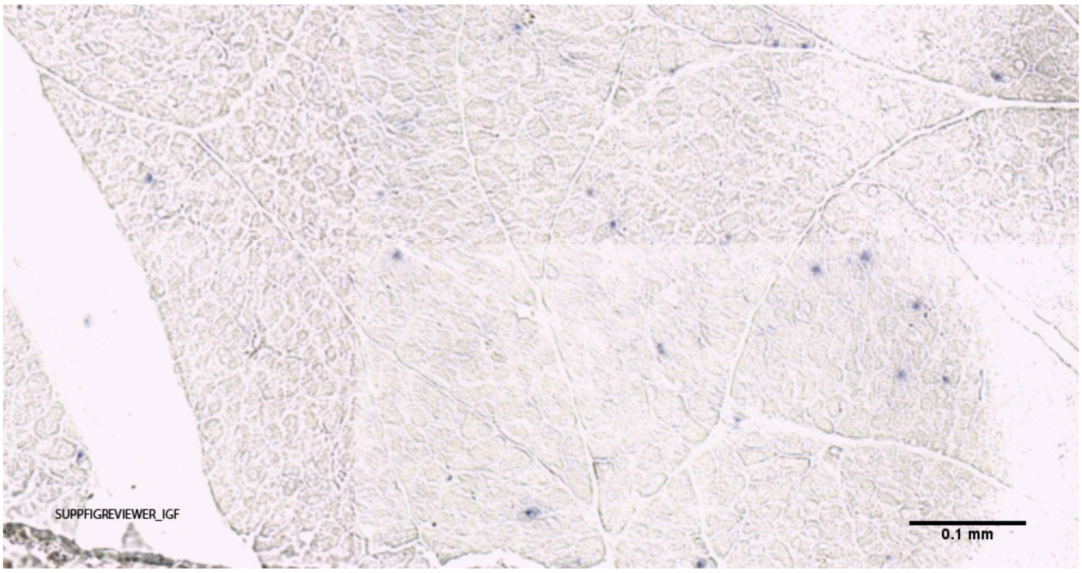

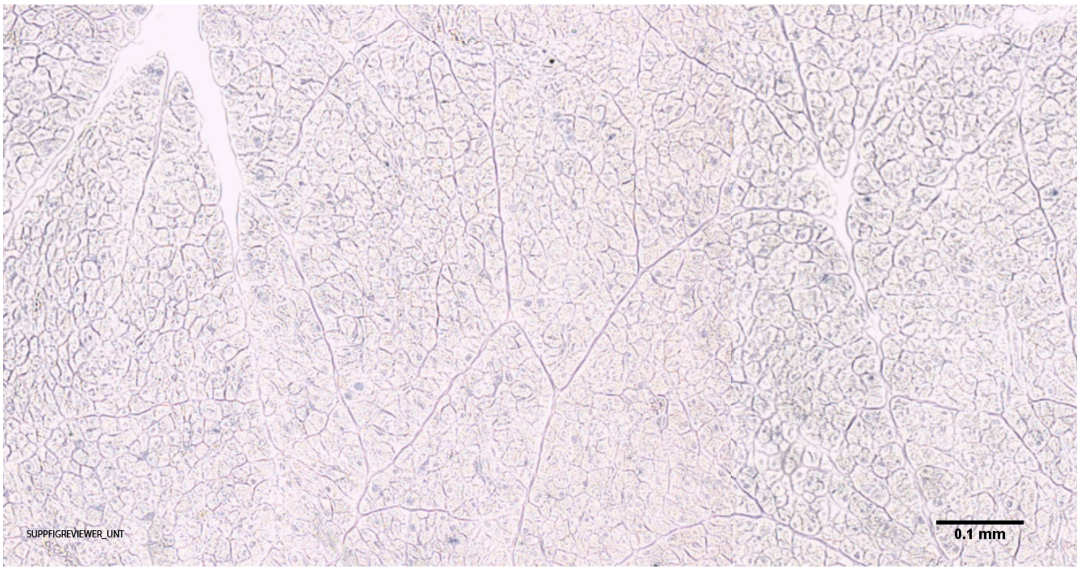

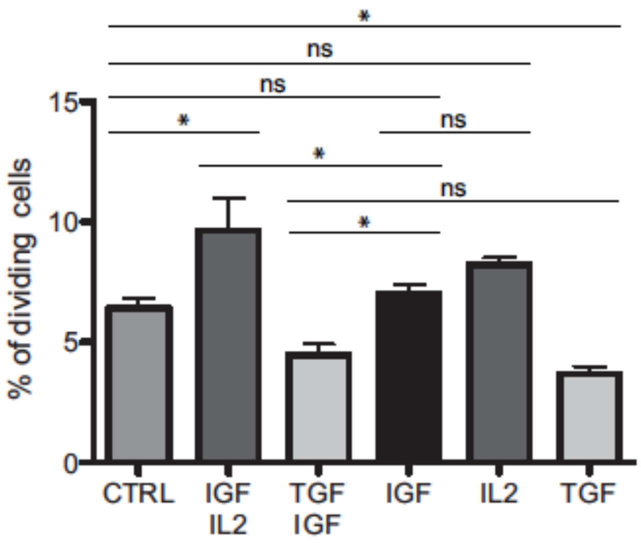

SuppFigReviewer_IL2. CFSE assay on CD4+CD25+ cells showing a cooperative stimulatory effect of IGF-1 and IL-2, and an inhibitory effect of TGF-ß in the proliferation of this Treg cell containing subset after 2 days of in vitro culture.

2nd Editorial Decision 07 March 2014

Thank you for the submission of your revised manuscript to EMBO Molecular Medicine. We have now received the enclosed reports from the referees that were asked to re-assess it. As you will see, while referee 1 is now supportive of publication, referee 3 remains concerned. I would like to give you another chance to fully satisfy this referee by replying satisfactorily to her/his comments. We do believe that this referee has a valid point as the conditional mouse model is critical for the study. As such I would appreciate if you could provide the information requested, both for the figures and materials and methods.

Please submit your revised manuscript as soon as possible.

In case your manuscript would then be accepted, could you please provide the following:

Every paper now includes a 'Synopsis' to further enhance their discoverability. Synopses are displayed on the html and they are freely accessible to all readers. They include a short standfirst - written by the editor- as well as 2-5 one sentence bullet points that summarise the paper. These should be complementary to the abstract - i.e. not repeat the same text. We encourage inclusion of key acronyms and quantitative information. Please use the passive voice.
Could I ask you to provide the bullet points in a separate file/via email please?

I look forward to reading a new final revised version of your manuscript. Please note that the manuscript may be sent back to the referee 2. For this reason I would strongly advise against sending back an incomplete revision that would not fully satisfy this reviewer.

***** Reviewer's comments *****

Referee #1 (Remarks):

I am satisfied with the revision, and this manuscript is now suitable for publication in EMBO.

Referee #3 (Remarks):

This is an improved manuscript, showing interesting effects of IGF1 on Treg in vitro and in vivo. Where statements about Treg enrichment in vivo are made, the data showing % Treg of CD4 cells and % CD4 cells is shown, supporting the claims (Figs 4 and S2). However, this is still not the case for the conditional knockout experiment, the most important part of this work. In the contact hypersensitivity experiment, only the # of Foxp3+ cells per spleen is shown (Fig 6C). It is not possible to conclude anything without showing % Foxp3+ cells among CD4+ T cells, and # of CD4 T cells per spleen. Also, the text mentions "proliferative status" in this experiment. Proliferative status should be shown as % Ki67+ of Foxp3+ cells. For this data set (contact hypersensitivity experiment), the same genetic controls shown in Fig S3A-C should be shown for the percentages and cell numbers requested. Furthermore, the Methods state that "Direct proliferative stimulation of Treg cells by rhIGF-I in vivo was determined by Foxp3 expression analysis of sorted CD4 positive cells from Igf1rfl/flFoxp3cre spleens in the context of a contact hypersensitivity response," however rhIGF1 isn't used in this experiment as described in the Results and Figures. This is confusing.

Overall, more experimental detail should be communicated, particularly in figure legends.

1. In Fig 1 and 2 legends, please state that CD3/CD28 stimulation was performed, and for each section of Fig 2, state if human our mouse cells are used.
2. In Fig 2C, should the first column be labeled IGF-1 + Inhibitor?
3. In Fig 2F, the data is processed too much. % inhibition of what? What is the readout for proliferation?
4. All CD4+ T cells should be shown in Fig 6A and S3D, not just Foxp3+ cells, to demonstrate similar background staining for Foxp3- cells.
5. The IGF1R Ab used in the western blot is not described. Please add this to the Methods section.
6. For Fig 6, should it read...
"Lanes 3, 4: activated CD4+CD25-CD44+CD62L-; Lanes 5, 6: resting CD4+CD25-CD44-CD62L-"
Rather than...
"Lanes 3, 5: activated CD4+CD25-CD44+CD62L-; Lanes 4, 6: resting CD4+CD25-CD44-CD62+-"
In the text referring to this western blot, it may be more clear to describe the cells as naive and

memory. As written it may imply that T cells were activated in vitro.
7. Fig S2F should be gated on CD4+ T cells so that the values correspond to S2G.
8. Fig S1C should be annotated more so that it is clear what all the plots represent.
9. Fig 5C: Fold change of what measurement?
10. What experiment does Fig S3H refer to? It is not mentioned in the Results section.

2nd Revision - authors' response 14 July 2014

Referee comment: *This is an improved manuscript, showing interesting effects of IGF1 on Treg in vitro and in vivo.*

Where statements about Treg enrichment in vivo are made, the data showing % Treg of CD4 cells and % CD4 cells is shown, supporting the claims (Figs 4 and S2). However, this is still not the case for the conditional knockout experiment, the most important part of this work. In the contact hypersensitivity experiment, only the # of Foxp3+ cells per spleen is shown (Fig 6C). It is not possible to conclude anything without showing % Foxp3+ cells among CD4+ T cells, and # of CD4 T cells per spleen.

Also, the text mentions "proliferative status" in this experiment. Proliferative status should be shown as % Ki67+ of Foxp3+ cells. For this data set (contact hypersensitivity experiment), the same genetic controls shown in Fig S3A-C should be shown for the percentages and cell numbers requested.

Response: The additional conditional knockout data requested above have been now incorporated in Fig. 6C and Fig. S3J-K and Fig. S4A-C. This includes new Foxp3 data and analysis with additional genetic controls, which show decreased Treg cell numbers in CKO peripheral blood (Fig. 6C and Fig. S3J-K) supporting the role of the IGF-1 axis in maintaining Treg cell numbers. As suggested, Fig. 6D and Fig. S4B show the proliferative status as % Ki67+ of Foxp3+ cells in the spleen. The rest of the spleen data and analysis has been moved to supplementary material (Fig. S4A-C). Unfortunately, lack of a sufficiently large mouse cohort prevented the repetition of this experiment with all the controls requested. However, given the fact that no differences were ever observed in any of the experiments performed among the different control mice groups (Fig. 6, Fig. S3 and Tables S4 and S5) we believe that the results presented are valid and should be included.

Referee comment: *Furthermore, the Methods state that "Direct proliferative stimulation of Treg cells by rhIGF-I in vivo was determined by Foxp3 expression analysis of sorted CD4 positive cells from Igf1rfl/flFoxp3cre spleens in the context of a contact hypersensitivity response," however rhIGF1 isn't used in this experiment as described in the Results and Figures. This is confusing.*

Response: This has been corrected. It now reads: "Direct proliferative stimulation of Treg cells by IGF-I *in vivo* was determined by flow cytometric analysis of CD4 positive cells from *Igf1r*$^{fl/fl}$*Foxp3*^{cre} spleens in the context of a contact hypersensitivity response".

Referee comment *Overall, more experimental detail should be communicated, particularly in figure legends.*

1. In Fig 1 and 2 legends, please state that CD3/CD28 stimulation was performed, and for each section of Fig 2, state if human our mouse cells are used.

Response: The suggested changes have been introduced:

Fig 1B legend now reads: "Control and treated cells were stimulated with anti-CD3 and CD28 antibodies".

Fig 2A legend now reads: "In all *in vitro* experiments, cells were stimulated with anti-CD3 and CD28 antibodies".

Throughout Fig 2 legend, it is now stated that murine cells were used.

Referee comment: *In Fig 2C, should the first column be labeled IGF-1 + Inhibitor?*

Response: This has been changed in the Figure as suggested.

Referee comment: *In Fig 2F, the data is processed too much. % inhibition of what? What is the readout for proliferation?*

Response: The percent inhibition refers to the number of Foxp3$^+$ cells in cultures treated with IGF-1) normalized to the background (i.e., number of Foxp3$^+$ cells in untreated control cultures without IGF-1). We used percent inhibition as a measure to fairly compare different experiments.

For clarity, we have moved those results to Fig.S2B and included in Fig. 2F an additional example of a similar experiment, where we analyzed the effect of the same inhibitors on proliferation (i.e., Ki67$^+$ cells amongst Foxp3$^+$ cells). Now Fig 2F legend reads as follows: "Flow cytometric analysis showing the effect of inhibitors of AKT (Deguelin, 1 µM; Deg), PI-3 kinase (Ly-294,002, 10 µM; LY), and MAPK (PD.98,059, 10 µM; PD) on the proliferation of murine Treg cells after two day treatment with rhIGF-1 (*P<0.05; n=2)". An explanatory note has been added in Fig. S2B legend: "Percentage of inhibition in Foxp3+ cell numbers with respect to the IGF-1 stimulatory effect (i.e., number of Foxp3+ cells in samples treated with IGF-1) normalized to the background (i.e., number of Foxp3+ cells in untreated controls, no IGF-1 addition)".

Referee comment: *All CD4+ T cells should be shown in Fig 6A and S3D, not just Foxp3+ cells, to demonstrate similar background staining for Foxp3- cells.*

Response: This has been changed as suggested. Now Fig. 6A and S3H (previously S3D) show all CD4$^+$ cells and demonstrate a similar background for Foxp3$^-$ cells.

Referee comment: *The IGF1R Ab used in the western blot is not described. Please add this to the Methods section.*

Response: This description has been added in the Methods section (page 15). It now reads: "For the Western blot analysis, a rabbit mAb IGF-I Receptor β (D23H3) XP® (Cell Signaling Technology) was used".

Referee comment: *For Fig 6, should it read... "Lanes 3, 4: activated CD4+CD25-CD44+CD62L-; Lanes 5, 6: resting CD4+CD25-CD44-CD62L-)" Rather than... "Lanes 3, 5: activated CD4+CD25-CD44+CD62L-; Lanes 4, 6: resting CD4+CD25-CD44-CD62+-)"*

In the text referring to this western blot, it may be more clear to describe the cells as naive and memory. As written it may imply that T cells were activated in vitro.

Response: The legend has been corrected following the suggestion and now it reads: "IGF-1 receptor western blot analysis of different populations of CD4 positive cells from *Igf1r$^{fl/fl}$* (CTRL) and *Foxp3^{cre} Igf1r$^{fl/fl}$* (KO) mice showing specific deletion of the IGF-1 receptor in the CKO Treg population. Lanes 1, 2: Treg cells (CD4$^+$CD25^{high}), Lanes 3, 5: memory CD4$^+$CD25$^-$CD44$^+$CD62L$^-$ T cells; Lanes 4, 6: naive CD4$^+$CD25$^-$CD44$^-$CD62$^+$ T cells Bottom panel: Loading controls (ß-

actin)".

Referee comment: *Fig S2F should be gated on CD4+ T cells so that the values correspond to S2G.*

Response: Cells in the plot shown in Fig. S2F (now Fig. S2G) are gated on $CD4^+$. Statistical analysis is shown in the figure and not in S2G as the previous legend stated. Previous Fig. S2G (now Fig. S2I) has been now correctly labeled and corresponds to the experiment shown in Fig. S2H.

Referee comment: *Fig S1C should be annotated more so that it is clear what all the plots represent.*

Response: The figure has been further annotated to better explain the different plots.

Referee comment: *Fig 5C: Fold change of what measurement?*

Response: As stated in the new version, it refers to the fold change in the number of $Foxp3^+$ cells in the spinal cord compared to control.

Referee comment: *What experiment does Fig S3H refer to? It is not mentioned in the Results section.*

Response: Fig. S3H, now Fig. S4F, refers to a statement made in the first paragraph of the Materials and Methods section (*in vivo* experiments) on page 13: "Control mice were either sham-operated mice or implanted with PBS (solvent) delivering pumps, as no difference was observed between these two groups compared to rhIGF-1 delivering pumps (Fig S4F)".

Editorial Decision 15 July 2014

Thank you for the submission of your revised manuscript to EMBO Molecular Medicine. After carefully evaluating your revised article, I am pleased to inform you that we will be able to accept your manuscript pending editorial final amendments:
