# Supplementary material for: Insulin-like growth factor-1 stimulates regulatory T cells and suppresses autoimmune disease
Source: EMBO Mol Med. 2014 Nov 3;6(11):1423–35. doi: 10.15252/emmm.201303376 (PMC4237469; doi:10.15252/emmm.201303376)
Supplement: Supplementary file 5 [file emmm0006-1423-sd5.pdf]

SUPPORTING INFORMATION

SUPPLEMENTARY FIGURE LEGENDS

Figure S1.

A. Sorting strategy for human Treg cell isolation. $CD4^{+}$ $CD25^{+}$ $CD127^{low}$ cells were sorted from human peripheral blood as indicated.

B. Fold expansion of proliferating (Ki67+; $P<0.05$; n=3) Treg cells after a 2 day stimulation of FACS sorted CD4+CD25+ cells with rhIGF-1.

C. Representative example of flow cytometric analysis showing that naive Treg cells retain their ability to suppress T effector (Teff) cell proliferation in vitro after rhIGF-1 treatment (ratios Treg:Teff, from top to bottom 1:2, 1:4, 1:8, 1:16, 1:32, 1:64).

D. Flow cytometric apoptotic assay showing no difference in the distribution of Annexin V and living dye (CD4+CD25+) stained populations (containing Treg cells) (Live: Annexin V- Living dye-, Early apoptotic: Annexin V+ Living dye, Late apoptotic: Annexin V+ Living dye+, Necrotic: Annexin V- Living dye+; P=0.996; n=2).

E. Representative flow cytometric analysis of surface CD71 expression induced by rhIGF-1 on the Treg containing (CD4+ CD25+) subset shows modest but consistent upregulation of this cell proliferation marker.

Figure S2.

A. Flow cytometric analysis of surface CD71, CD44 and CD62L expression induced by rhIGF-1 on the Treg containing ($CD4^+$ $CD25^+$) subset shows different sensitivity to inhibitors of AKT (Deguelin, 1 μM), PI-3 kinase (Ly-294,002, 10 μM), and MAPK (PD.98,059, 10 μM) on Treg cells after 2 day treatment with rhIGF-1 (*P<0.05; n=2).

B. As in Fig. 2F, flow cytometric analysis showing the sensitivity to inhibitors of AKT (Deguelin, 1 μM; Deg), PI-3 kinase (Ly-294,002, 10 μM; LY), and MAPK (PD.98,059, 10 μM; PD) on murine Treg cell proliferation after two day treatment with rhIGF-1. Percentage of inhibition in $Foxp3^+$ cell numbers with respect to the IGF-1 stimulatory effect (i.e., number of $Foxp3^+$ cells in samples treated with IGF-1) normalized to the background (i.e., number of Foxp3+ cells in untreated controls, no IGF-1 addition).

C. Human rIGF-1 was determined in peripheral blood by ELISA at the indicated times after surgical implantation of the rhIGF-1 minipump and compared with either untreated mice (P5d=0.002, n=19; P20d=0.028; n=8) or mice treated with STZ (CTRL; P=0.031; n=9).

D. Intraperitoneal glucose tolerance test (GTT) performed three weeks from the first STZ injection and four weeks after surgical implantation of the rhIGF-1 minipump shows that IGF-I treatment ameliorates glucose response after STZ treatment (P<0.05).

E. Determination of the percentage of insulin stained area in the pancreatic tissue at day 97 in untreated (UNT) and after STZ treatment in rhIGF-1 treated (IGF-I) and control mice (CTRL; P=0.128; n=9).

F. GTT was performed at the indicated time points and the area under the curve was calculated showing that rhIGF-1 treatment had no significant effect in non-diabetic mice (UNT IGF-I) compared to untreated mice (UNT; n=11).

G. Representative example of flow cytometric analysis of peripheral blood one week after implantation of rhIGF-1 minipumps shows increased $FoxP3^+$

(Treg; P=0.011, n=13) and KI67^{+} (Treg Ki67; P=0.007) Treg cells in STZ-treated mice.

H. Representative example of flow cytometric analysis showing that rhIGF-1 treatment increases the number of Treg cells ($CD4^{+}CD25^{+}Foxp3^{+}$) in the spleen of diabetic mice compared to control untreated or STZ-treated animals.

I. Flow cytometric analysis corresponding to figure S2H (*$P<0.05$; n=14).

J. Flow cytometric analysis showing that rhIGF-1 treatment increases the number of Treg cells ($CD4^{+}CD25^{+}$) in the spleen of diabetic mice compared to control untreated or STZ-treated animals (*$P<0.05$; n=14).

K. rhIGF-1 treatment has no significant effect on the total number of CD4+ cells in peripheral blood of STZ- treated animals one week after implantation of rhIGF-1 minipumps (n=13).

L. rhIGF-1 treatment decreases the total number of CD4+ cells in the spleen compared to control untreated (UNT) or STZ-treated mice (CTRL;*$P<0.05$; n=14).

M. Basal glucose levels after fasting indicate that IGF-1 treatment ameliorates diabetes progression in NOD mice (P33w=0.126, P39w=0.133; n =15).

Figure S3.

A-C. Mice treated with rhIGF-1 and $MOG_{35\text{-}55}$ peptide as in Fig. 5A were injected i.p. with either control or anti-CTLA-4 IgG, which abrogated both clinical improvement at day 13 (A; P=0.037; n=15) and decreased Foxp3 infiltrating cells in the spinal cord induced by rhIGF-1 treatment (B and C; P=0.014; n=15).

D. PCR of genomic DNA from *Igf1r*$^{fl/fl}$*, Foxp3*^{cre} *and Foxp3*^{cre} *Igf1r*$^{fl/fl}$ mice confirms amplicon corresponding to exon 7 deletion in $CD4^+CD25^+Foxp3^+$ cells only in *Foxp3*^{cre} *Igf1r*$^{fl/fl}$ mice

E. Representative histograms of *Foxp3*^{cre} *Igf1r*$^{fl/fl}$ (KO), *Igf1r*$^{fl/+}$ and *Igf1r*$^{fl/fl}$ (CTRL) peripheral blood $CD4^+$ flow cytometric analysis showing no significant difference in the relative number of the $CD4^+Foxp3^+$ Treg containing subpopulation.

F. Flow cytometric analsysis corresponding to figure E (n=20).

G. Flow cytometric analysis of *Foxp3*^{cre} *Igf1r*$^{fl/fl}$ (KO), *Igf1r*$^{fl/+}$ and *Igf1r*$^{fl/fl}$ (CTRL) peripheral blood showing no significant difference in the number of $CD4^+$ cells (n=20).

H. Representative example of flow cytometric analysis showing that IGF-1 receptor-deficient Treg cells (*Foxp3*^{cre} *Igf1r*$^{fl/fl}$; CKO) express similar levels of Foxp3 compared to control cells (CTRL; n=24) in unchallenged conditions.

I. Flow cytometric analysis showing mean fluorescence intensity corresponding to Fig. S3H.

J. Flow cytometric analysis of peripheral blood from contact-hypersensitized *Foxp3*^{cre} *Igf1r*$^{fl/fl}$ (KO), *Igf1r*$^{fl/+}$ and *Igf1r*$^{fl/fl}$ (CTRL) mice showing no significant difference in the number of $CD4^+$ cells.

K. Representative histograms of *Foxp3*^{cre} *Igf1r*$^{fl/fl}$ (KO), *Igf1r*$^{fl/+}$ and *Igf1r*$^{fl/fl}$ (CTRL) peripheral blood $CD4^+$ flow cytometric analysis corresponding to Fig.

6C, and showing decreased Foxp3$^+$ Treg cells in contact-hypersensitized *Foxp3^{cre} Igf1r$^{fl/fl}$* mice.

Figure S4.

A. Flow cytometric analysis shows reduced total splenic Treg cell numbers (Treg; P=0.002, n=16) and proliferating Treg cells (Treg Ki67; P=0.001) in contact-hypersensitized Foxp3^{cre} Igf1r$^{fl/fl}$ mice.

B. Flow cytometric analysis corresponding to figure 6D. *P<0.05

C. Flow cytometric analysis shows no difference in the percentage of CD4^{+} cells between contact-hypersensitized wildtype and Foxp3^{cre} Igf1r$^{fl/fl}$ mice.

D. Gating strategy of the 3 subpopulations of *Foxp3^{cre} Igf1r$^{fl/}$fl* splenic CD4^{+} positive cells showed in Fig. S4E. Fluorescence minus one (FMO) control corresponding to CD25 is shown on the right panel.

E. Flow cytometric analysis of GFP/Cre expression (driven by the Foxp3 promoter) in 3 subpopulations of *Foxp3^{cre} Igf1r$^{fl/}$fl* splenic CD4^{+} cells (CD25^{high}, CD25^{int} and CD25^{neg}) showing that GFP/Cre is expressed exclusively in CD25^{+} cells and enriched in CD25^{high}.

F. Increased proliferating Treg cells in peripheral blood one week after the implantation of IGF-I pumps (pump IGF-I; *P<0.05) compared to sham operated animals (sham) or implantation of PBS (solvent) pumps (pump).

FIG. S1

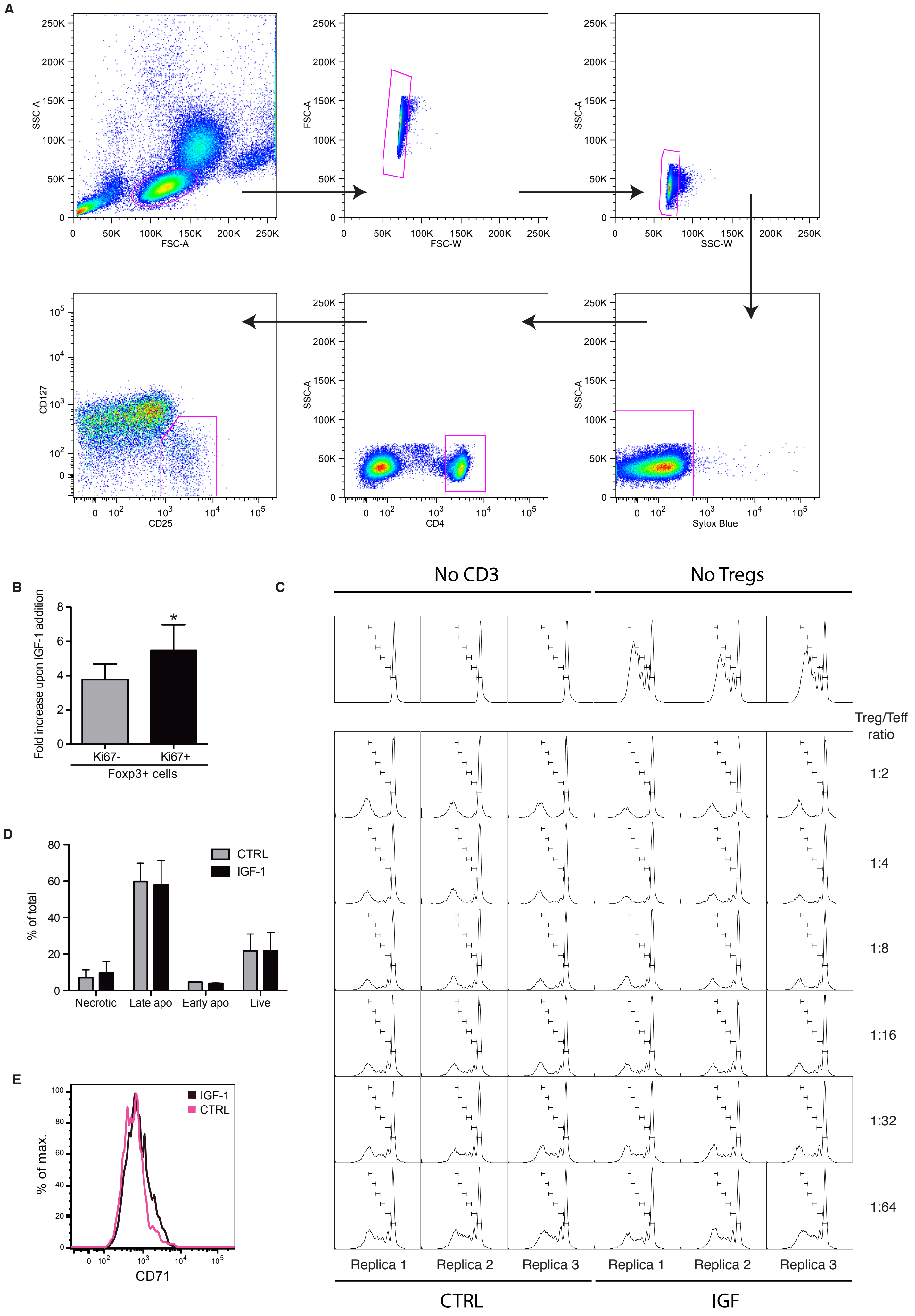
A
SSC-A
FSC-A
FSC-W
SSC-W
Sytox Blue
CD4
CD127
CD25
B
Fold increase upon IGF-1 addition
Ki67-
Ki67+
Foxp3+ cells
C
No CD3
No Tregs
Treg/Teff ratio
1:2
1:4
1:8
1:16
1:32
1:64
Replica 1
Replica 2
Replica 3
Replica 1
Replica 2
Replica 3
CTRL
IGF
D
% of total
CTRL
IGF-1
Necrotic
Late apo
Early apo
Live
E
% of max.
IGF-1
CTRL
CD71

FIG. S2

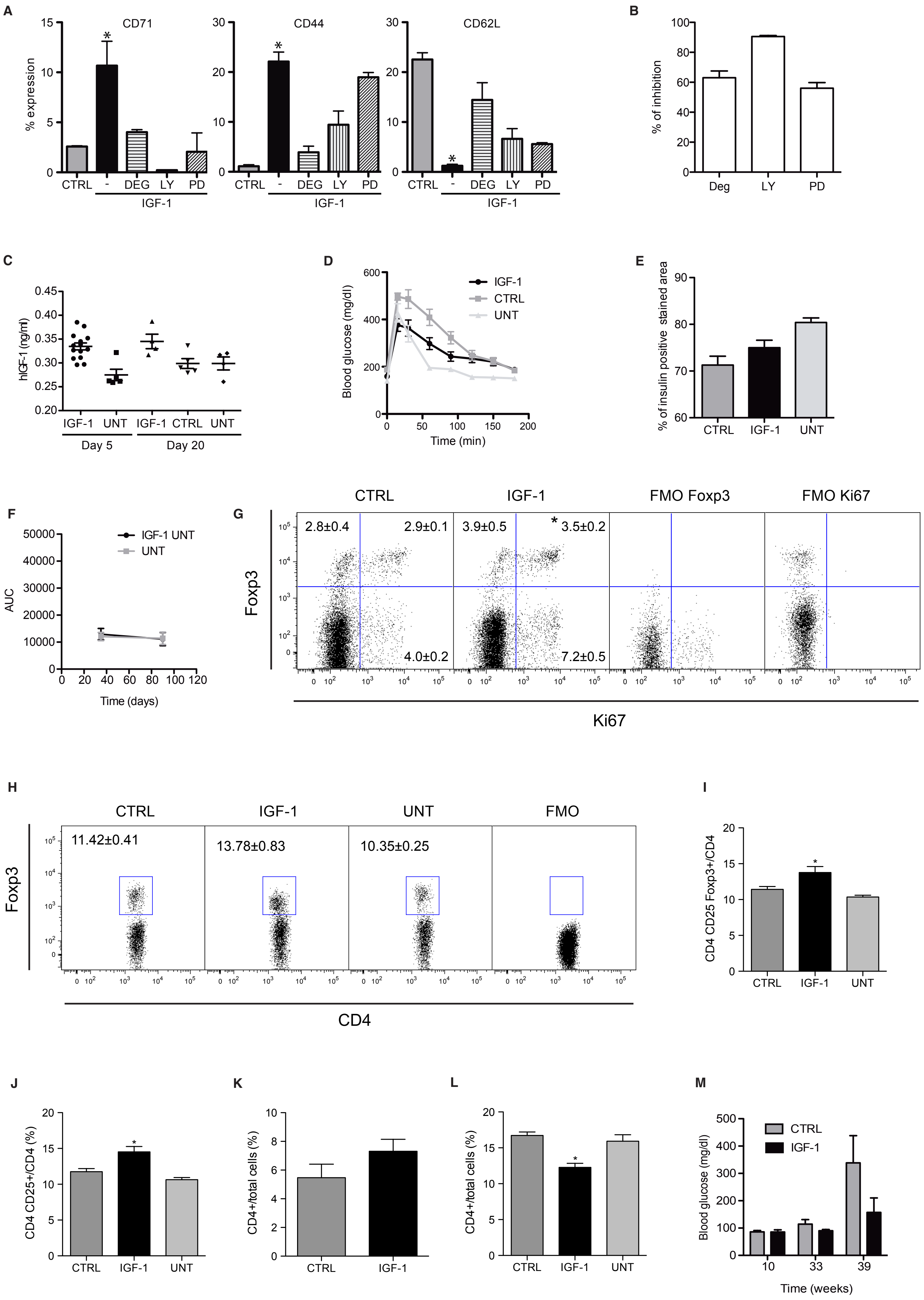
A
CD71
CD44
CD62L
% expression
CTRL - DEG LY PD
IGF-1
B
% of inhibition
Deg LY PD
C
hIGF-1 (ng/ml)
IGF-1 UNT IGF-1 CTRL UNT
Day 5
Day 20
D
Blood glucose (mg/dl)
IGF-1
CTRL
UNT
Time (min)
E
% of insulin positive stained area
CTRL IGF-1 UNT
F
AUC
IGF-1 UNT
UNT
Time (days)
G
CTRL
IGF-1
FMO Foxp3
FMO Ki67
Foxp3
2.8±0.4
2.9±0.1
4.0±0.2
3.9±0.5
3.5±0.2
7.2±0.5
Ki67
H
CTRL
IGF-1
UNT
FMO
Foxp3
11.42±0.41
13.78±0.83
10.35±0.25
CD4
I
CD4 CD25 Foxp3+/CD4
CTRL IGF-1 UNT
J
CD4 CD25+/CD4 (%)
CTRL IGF-1 UNT
K
CD4+/total cells (%)
CTRL IGF-1
L
CD4+/total cells (%)
CTRL IGF-1 UNT
M
Blood glucose (mg/dl)
CTRL
IGF-1
Time (weeks)

FIG. S3

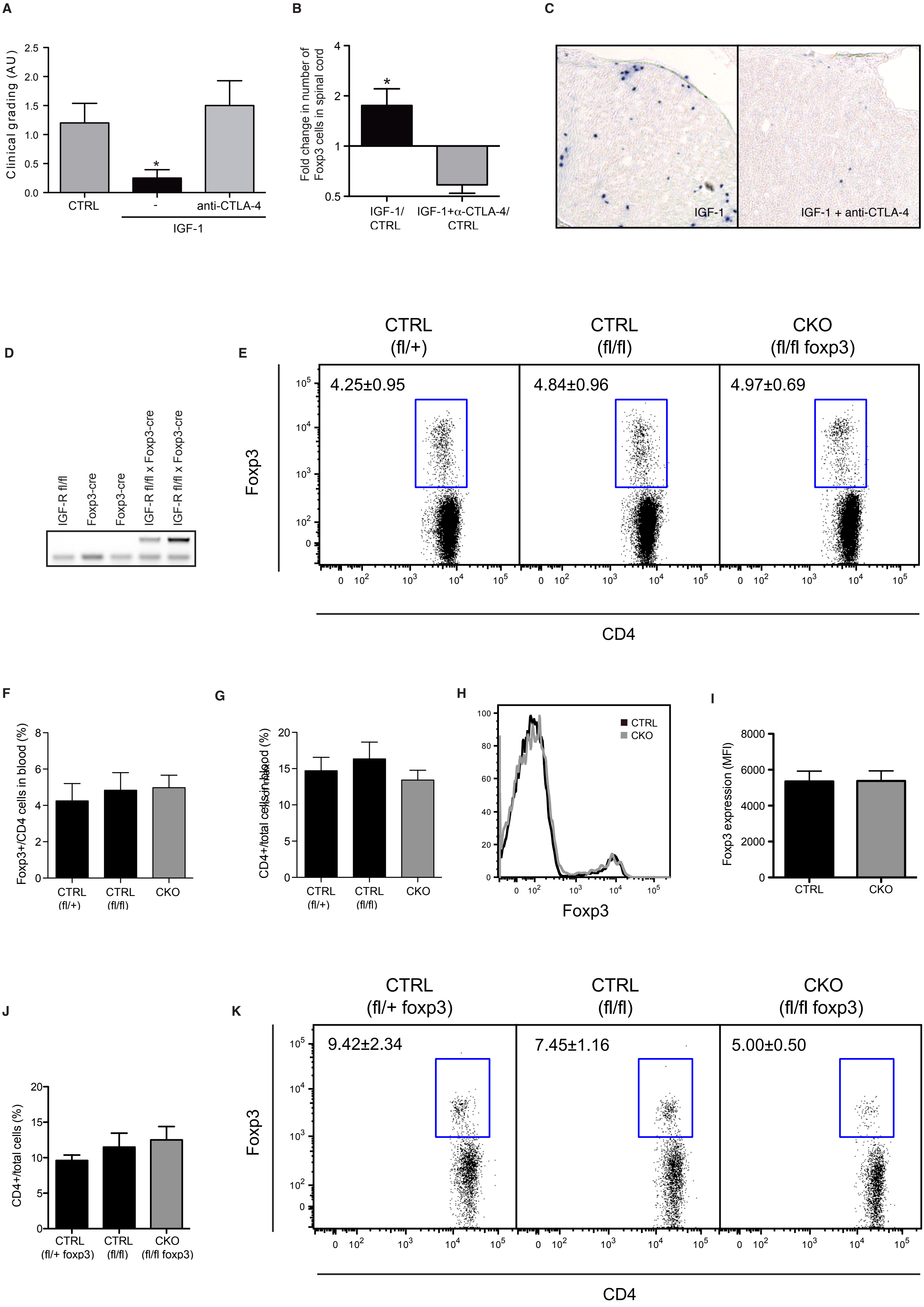
A
Clinical grading (AU)
2.5
2.0
1.5
1.0
0.5
0.0
*
CTRL
-
anti-CTLA-4
IGF-1
B
Fold change in number of Foxp3 cells in spinal cord
4
2
1
0.5
*
IGF-1/ CTRL
IGF-1+α-CTLA-4/ CTRL
C
IGF-1
IGF-1 + anti-CTLA-4
D
IGF-R fl/fl
Foxp3-cre
Foxp3-cre
IGF-R fl/fl x Foxp3-cre
IGF-R fl/fl x Foxp3-cre
E
CTRL (fl/+)
CTRL (fl/fl)
CKO (fl/fl foxp3)
4.25±0.95
4.84±0.96
4.97±0.69
Foxp3
CD4
F
Foxp3+/CD4 cells in blood (%)
CTRL (fl/+)
CTRL (fl/fl)
CKO
G
CD4+/total cells in blood (%)
CTRL (fl/+)
CTRL (fl/fl)
CKO
H
CTRL
CKO
Foxp3
I
Foxp3 expression (MFI)
8000
6000
4000
2000
0
CTRL
CKO
J
CD4+/total cells (%)
CTRL (fl/+ foxp3)
CTRL (fl/fl)
CKO (fl/fl foxp3)
K
CTRL (fl/+ foxp3)
CTRL (fl/fl)
CKO (fl/fl foxp3)
9.42±2.34
7.45±1.16
5.00±0.50
Foxp3
CD4

FIG. S4

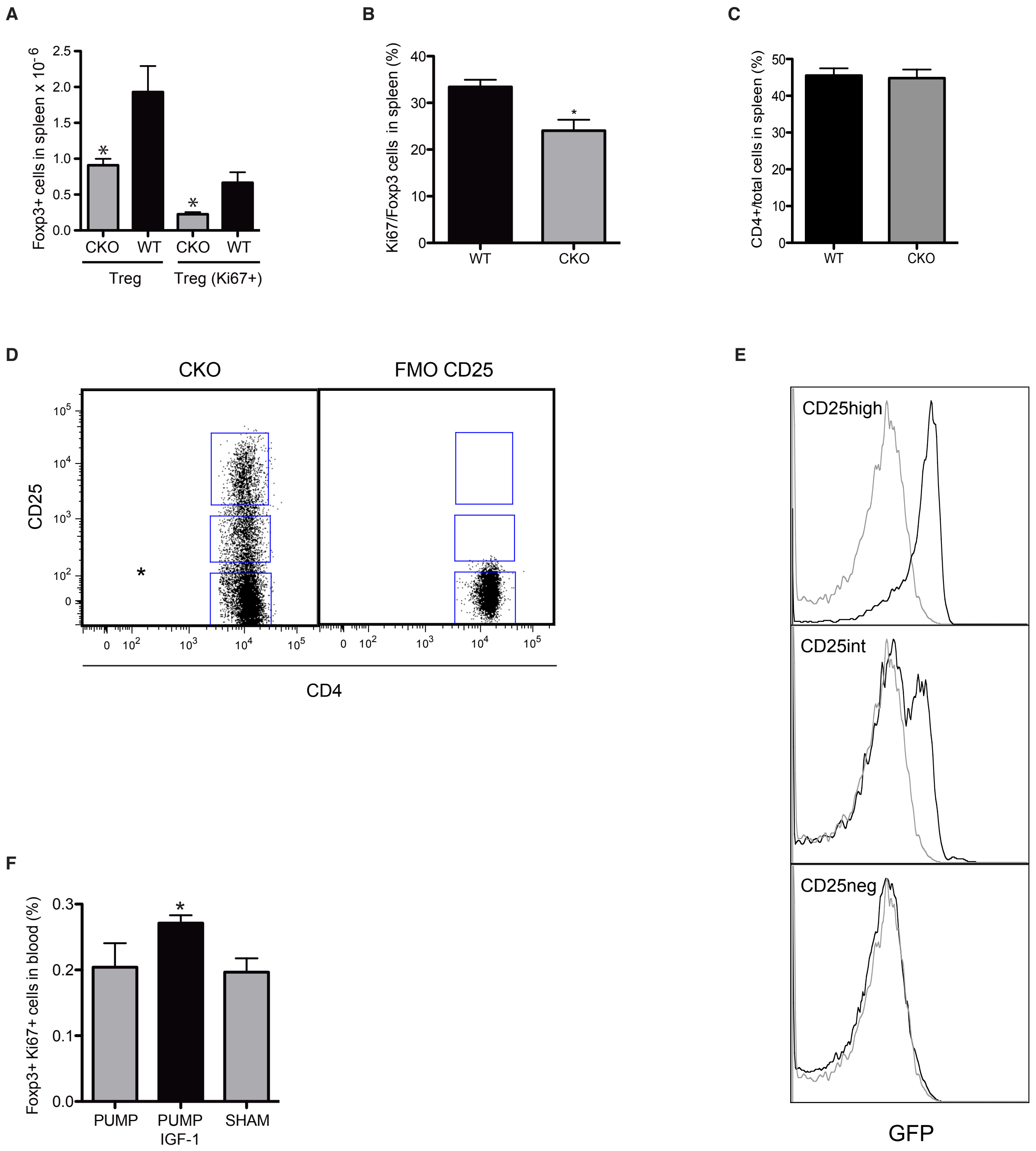
A
Foxp3+ cells in spleen x 10-6
2.5
2.0
1.5
1.0
0.5
0.0
CKO
WT
CKO
WT
Treg
Treg (Ki67+)
B
Ki67/Foxp3 cells in spleen (%)
40
30
20
10
0
WT
CKO
C
CD4+/total cells in spleen (%)
50
40
30
20
10
0
WT
CKO
D
CKO
FMO CD25
CD25
CD4
E
CD25high
CD25int
CD25neg
GFP
F
Foxp3+ Ki67+ cells in blood (%)
0.3
0.2
0.1
0.0
PUMP
PUMP IGF-1
SHAM

TABLE S1
Genes differentially expressed (>2X) in Treg cells upon rhIGF-I stimulation.

TABLE S2
Genes differentially expressed (>2X) in Treg cells upon rhIGF-I stimulation (IGF) that overlap with the Treg signature.
Gene expression analysis in Treg cells stimulated by IGF-1 of Treg signature genes and clusters 1 to 7 as defined by Hill et al., 2007.

TABLE S3
Transcription target enrichment analysis of genes upregulated upon IGF-1 stimulation of Treg cells.

TABLE S4
Peripheral blood analysis of CTRL ($Igf1r^{fl/fl}$) and KO mice ($Foxp3^{cre}$ $Igf1r^{fl/fl}$).

TABLE S5
Contact hypersensitivity assay in control mice.

Table S4

Leukocytes	CTRL	KO
**WBC**	4.85±1.07	6.09±2.38
**NE**	1.04±0.51	1.42±0.63
**LY**	3.52±1.21	4.38±2.09
**MO**	0.20±0.07	0.23±0.06
**EO**	0.06±0.07	0.05±0.03
**BA**	0.02±0.03	0.02±0.01
**Erythrocytes**		
**RBC**	8.30±0.54	8.99±0.84
**HB**	8.76±0.64	9.10±0.94
**HCT**	63.0±4.5	60.9±5.9
**MCV***	76.2±7.2	67.9±5.8
**MCH**	10.6±0.8	10.1±0.6
**MCHC**	14.2±1.8	15.1±1.6
**RDW**	16.7±0.6	17.2±0.7
**Thrombocytes**		
**PLT**	427±183	504±185
**MPV***	4.43±0.16	4.65±0.08

Peripheral blood analysis of CTRL ($Igf1r^{fl/fl}$) and KO aged (>1 year old) mice ($Foxp3^{cre}$ $Igf1r^{fl/fl}$). n=20

*P<0.05

Table S5

Genotype	Ear thickness
**Foxp3^{Cre} Igf1r$^{fl/fl}$**	52±9
**Igf1r$^{fl/fl}$**	54±6
**Foxp3^{Cre} Igf1r$^{+/fl}$**	48±2
**Igf1r$^{+/fl}$**	46±5

Ear thickness (10^{-2} mm) was measured after contact hypersensitivity reaction in the indicated genotypes. No significant difference was found in any of the comparisons (n=30).
